# The cryptonephridial/rectal complex: an evolutionary adaptation for water and ion conservation

**DOI:** 10.1111/brv.13156

**Published:** 2024-10-22

**Authors:** Robin Beaven, Barry Denholm

**Affiliations:** ^1^ Hugh Robson Building, George Square, Deanery of Biomedical Sciences The University of Edinburgh Edinburgh EH8 9XD UK

**Keywords:** renal system, cryptonephridium, insect, arthropod, beetle, Coleoptera, Tenebrionidae, *Tribolium castaneum*, countercurrent, arid environment

## Abstract

Arthropods have integrated digestive and renal systems, which function to acquire and maintain homeostatically the substances they require for survival. The cryptonephridial complex (CNC) is an evolutionary novelty in which the renal organs and gut have been dramatically reorganised. Parts of the renal or Malpighian tubules (MpTs) form a close association with the surface of the rectum, and are surrounded by a novel tissue, the perinephric membrane, which acts to insulate the system from the haemolymph and thus allows tight regulation of ions and water into and out of the CNC. The CNC can reclaim water and solutes from the rectal contents and recycle these back into the haemolymph. Fluid flow in the MpTs runs counter to flow within the rectum. It is this countercurrent arrangement that underpins its powerful recycling capabilities, and represents one of the most efficient water conservation mechanisms in nature. CNCs appear to have evolved multiple times, and are present in some of the largest and most evolutionarily successful insect groups including the larvae of most Lepidoptera and in a major beetle lineage (Cucujiformia + Bostrichoidea), suggesting that the CNC is an important adaptation. Here we review the knowledge of this remarkable organ system gained over the past 200 years. We first focus on the CNCs of tenebrionid beetles, for which we have an in‐depth understanding from physiological, structural and ultrastructural studies (primarily in *Tenebrio molitor*), which are now being extended by studies in *Tribolium castaneum* enabled by advances in molecular and microscopy approaches established for this species. These recent studies are beginning to illuminate CNC development, physiology and endocrine control. We then take a broader view of arthropod CNCs, phylogenetically mapping their reported occurrence to assess their distribution and likely evolutionary origins. We explore CNCs from an ecological viewpoint, put forward evidence that CNCs may primarily be adaptations for facing the challenges of larval life, and argue that their loss in many aquatic species could point to a primary function in conserving water in terrestrial species. Finally, by considering the functions of renal and digestive epithelia in insects lacking CNCs, as well as the typical architecture of these organs in relation to one another, we propose that ancestral features of these organs predispose them for the evolution of CNCs.

## I. INTRODUCTION

**Fig. 1:**
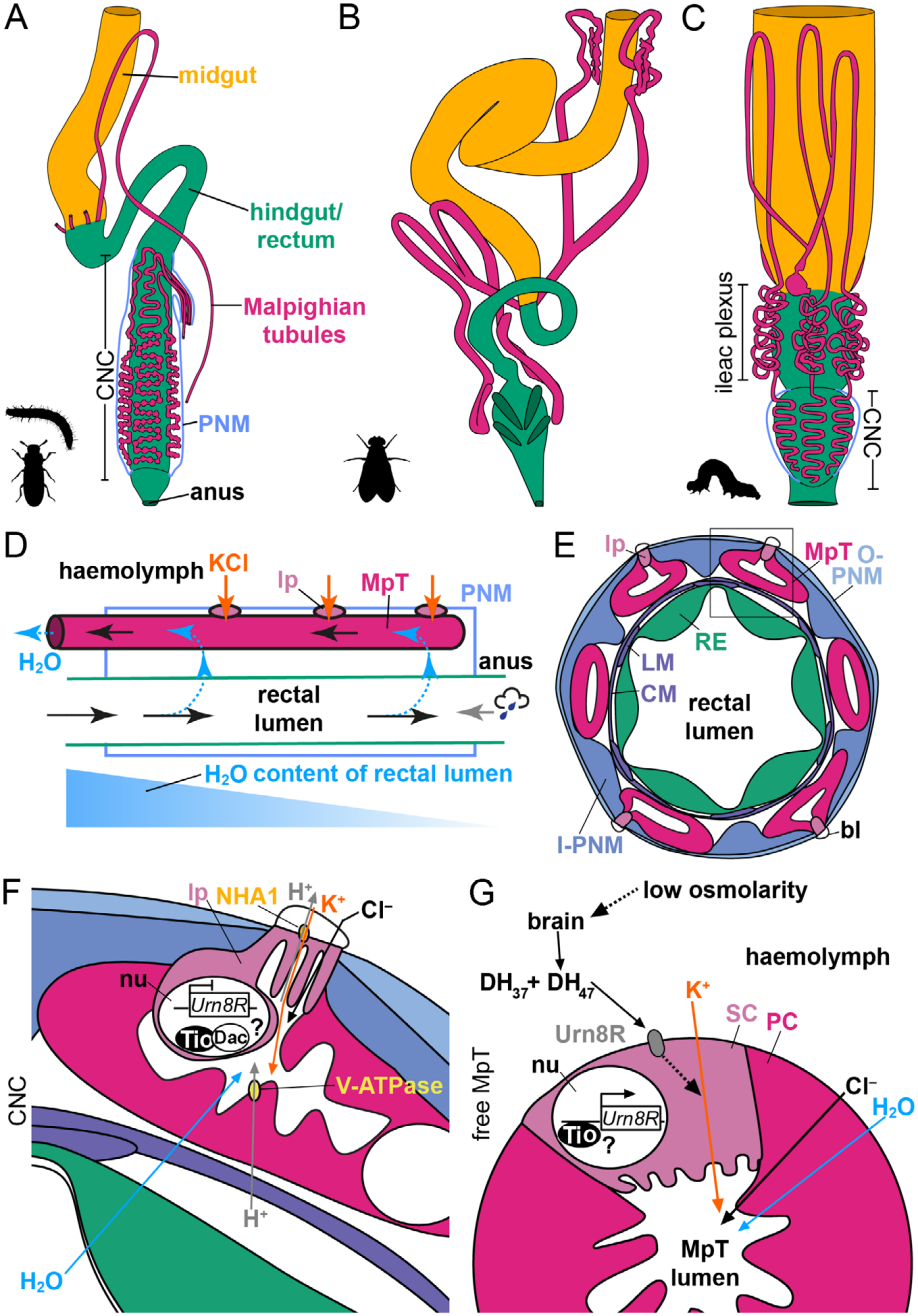
Cryptonephridial complex (CNC) organisation and function. (A) Schematic of gut and Malpighian tubule (MpT) organisation of *Tribolium castaneum* adult (a similar organisation is seen in the larval stage). The proximal, open ends of the MpTs insert into the boundary of the midgut and hindgut. As for many species of Coleoptera, *Tribolium* has a CNC, in which the distal segments of the MpTs lie on the external surface of the rectum, and are ensheathed with a perinephric membrane (PNM) tissue, a cross section of which is shown. All MpTs also have a region which lies freely in the haemolymph (part of one is shown for simplicity, compare with Fig. 4D). (B) In adult *Drosophila melanogaster*, MpTs are free within the haemolymph, and are not bound to the rectum (this is also the case in the larval stage). Similar organisations are found in diverse insects including most Diptera, and this is considered the ancestral state. (C) Larval *Trichoplusia ni* has a CNC organisation typical of most larval Lepidoptera, which has evolved independently from the CNCs of Coleoptera, but shows some remarkable similarities in organisation. Modified from O'Donnell & Ruiz‐Sanchez (2015). Generally in Lepidoptera species, the MpTs fold back on themselves within the CNC to form an inner and outer layer (Henson, 1937; Ishimori, 1924), not shown for simplicity. (D) A countercurrent organisation underpins the function of the CNC in beetles such as *Tribolium*. The system is insulated from water and ion exchange with the haemolymph by the PNM, with only the specialised leptophragma (lp) cells having access to the haemolymph. Potassium chloride is transported into the MpT lumen *via* the lp, driving a flux of water from the rectal contents into the MpTs. This fluid flows into the free portion of the MpTs where reabsorption of ions into the haemolymph can drive the return of water into the animal. The fluid flow in the rectum and MpTs are in opposite directions, meaning the osmolarity is highest at the posterior of the system (right), which maximises its ability to extract water, and minimises water loss from the animal. The system also enables the gain of water from humid air entering *via* the anus. (E) Schematic cross section through the CNC, as seen in *Tenebrio* and *Tribolium*. O‐ and I‐ PNM are the outer and inner layers, respectively, of the PNM. The lp of the MpTs lie beneath thin blisters (bl). Longitudinal and circular muscles (LM and CM) surround the rectal epithelium (RE). (F) A cross section of part of the CNC, corresponding to the boxed area in E. Vacuolar ATPase (V‐ATPase) pumps protons (H^+^) into the MpT lumen. Na^+^, K^+^/H^+^ antiporter 1 (NHA1) appears to act as a K^+^/H^+^ antiporter, using these protons to drive the transport of K^+^ into the MpT lumen *via* the lp. Along with Cl^−^, thought to flow along the electrochemical gradient established by K^+^ transport, this generates high osmolarity surrounding the rectum, drawing water from the rectal contents. The transcription factor, Dachshund (Dac), represses urinate receptor (Urn8R) expression, perhaps by binding and repressing another transcription factor, Tiptop (Tio), in the nucleus (nu). This may prevent the lp from increasing secretion in response to diuretic hormone 37 (DH_37_) and 47 (DH_47_) signalling. (G) Cross section through the free region of a MpT showing a secondary cell (SC) and principal cell (PC). SCs in this region express a hormone receptor (Urn8R), likely under control of the transcription factor Tio. In response to low osmolarity, the brain releases DH_37_ and DH_47_. These bind to the Urn8R receptor, upregulating K^+^ transport, and driving fluid secretion into the MpT lumen.

The cryptonephridial or rectal complex (CNC) is an organ architecture occurring in many insects that incorporates parts of the digestive and renal organs. It is remarkably prevalent and occurs in some of the largest and most evolutionarily successful insect groups including within Coleoptera and Lepidoptera (Fig. 1A–C), indicating its functional importance. The renal or Malpighian tubules (MpTs) are blind‐ending tubes opening into the gut, into which they expel fluid. In the ancestral condition in which there is no CNC (Fig. 1B), reabsorption of water and specific ions back into the animal occur in the MpT region closest to the gut (the proximal region of the MpT), and in the hindgut, particularly the rectum. This reabsorptive function is essential for animal homeostasis and therefore survival. Many insect species, and one myriapod, have been found to have a dramatic modification of this system, termed the CNC. Here, part of the MpTs (generally the distal portion) lie in intimate contact with the surface of the rectum, where the powerful secretory properties of the MpTs provide a highly efficient way to recover water and solutes from the luminal contents of the rectum. In some cases, CNCs provide a means to absorb water from vapour in the air (Fig. 1D). The phylogenetic distribution of CNCs suggests they have evolved independently multiple times, providing further indication of the functional importance of this adaptation.

The anatomy and physiology of CNCs have been studied over the past two centuries. More recently, the functioning of lepidopteran CNCs has been revealed down to a molecular level (Azuma *et al*., 2012; O'Donnell & Ruiz‐Sanchez, 2015; Audsley, Coast & Schooley, 1993; Liao, Audsley & Schooley, 2000; Moffett, 1994), and this work has been extensively discussed in a book chapter (Kolosov & O'Donnell, 2019*a*). Despite having been studied in great detail in classical physiological studies, the CNCs of tenebrionid beetles such as the mealworm *Tenebrio molitor* (hereafter *Tenebrio*), had remained unstudied in the age of molecular biology and advanced microscopy. With experimental advances in the model tenebrionid beetle, the red flour beetle *Tribolium castaneum* (hereafter *Tribolium*), including the sequencing of its genome (Herndon *et al*., 2020; Richards *et al*., 2008), establishment of molecular and genetic approaches (Gilles *et al*., 2019; Lorenzen *et al*., 2003; Trauner *et al*., 2009; Bucher, Scholten & Klingler, 2002; Tomoyasu *et al*., 2008; Gilles, Schinko & Averof, 2015), and imaging advances including live imaging (Benton, 2018; Benton, Akam & Pavlopoulos, 2013; Strobl & Stelzer, 2014; Strobl, Schmitz & Stelzer, 2015), *Tribolium* is now being used as a model species to advance the understanding of beetle CNC biology (King & Denholm, 2014; R. Beaven, K.V. Halberg & B. Denholm, in preparation; Naseem *et al*., 2023; Beaven *et al*., 2024b).

Here we review the current understanding of CNCs. We provide insights from tenebrionid beetles, including recent advances in molecular knowledge, as a point of focus. By relating to insights from diverse arthropod groups, we also seek to shed light more broadly on the functions of CNCs. Section II discusses CNC function and structure, along with physiology and endocrine control. Section III explores insights into the embryonic development of the CNC, which is beginning to inform our understanding of its evolution. Section IV looks at the distribution of CNCs in different arthropod groups, and the diversity of their structures, discussing the likely ecological and evolutionary significance of these organ systems.

## II. FUNCTION, STRUCTURE, PHYSIOLOGY, AND ENDOCRINOLOGICAL CONTROL OF THE CNC

### (1) CNC function

#### (a) Conserving and gaining water

Tenebrionid beetles can produce extremely dry faeces, as a result of the activity of their CNC in absorbing water and returning it to the haemolymph (Wigglesworth, 1932; Ramsay, 1964). This provides a powerful means to reduce water loss from the animal. Furthermore, starved *Tenebrio* larvae increase in body mass over time when kept in high‐humidity conditions (Buxton, 1930). This cannot be explained by gain from metabolism, so these animals appear to gain mass directly by absorbing atmospheric water vapour (Buxton, 1932). Water absorption occurs when the relative humidity is approximately 90% or higher (Machin, 1975; Hansen, Ramløv & Westh, 2004), and leads to an increase in larval water content, and a reduction in haemolymph osmolarity (Machin, 1975). Water vapour absorption in *Tenebrio* and other arthropod species requires specialised mechanisms, as the salt concentration within the animal is not able to account for absorption of water from the atmosphere, and would only lead to a net transfer of water into the animal at a relative humidity of 99% or higher (Buxton, 1932; Edney, 1957). Blocking the anus with wax prevents gain of water, demonstrating this to be the route of vapour entry (Machin, 1975; Dunbar & Winston, 1975; Noble‐Nesbitt, 1970).

It has been questioned whether uptake of water from the atmosphere by *Tenebrio* truly represents an adaptation, or is simply a by‐product of a mechanism for extraction of water from faeces (Dunbar & Winston, 1975). There is, however, a large increase in metabolic activity preceding water vapour uptake from air, as determined using calorimetric techniques. This suggests an actively regulated mechanism of water vapour uptake (Hansen *et al*., 2004). Furthermore in the tenebrionid beetles *Tenebrio* and *Onymacris marginipennis*, calculation of the rate of passive diffusion of water vapour into the lumen of the rectum show that it is insufficient to account for the water uptake observed, and some form of active ventilation is proposed to account for this (Coutchié & Machin, 1984). Such a mechanism would support water vapour absorption being an evolved adaptation.

The extent to which conserving water is a critical function of the CNCs of Lepidoptera has been less clear. Aquaporin water channels are expressed in the rectal epithelium, and the outer MpTs of the CNC in *Bombyx mori* larvae (Azuma *et al*., 2012). These likely underpin water transport from the rectal contents, and although this may be to drive other processes such as clearance of nitrogenous waste, it has been shown that the recovery of water from the rectal contents is under endocrine control (see references within Kolosov & O'Donnell, 2019*a*), suggesting its importance for osmotic homeostasis.

#### (b) Recycling of ions

It is possible that the CNC of tenebrionid beetles also serves to recycle solutes, although this has not been extensively studied in this group. In larval *Manduca sexta* and *Trichoplusia ni*, both lepidopterans with a CNC, evidence supports an important role of the CNC in regulating the animal's pH balance (Kolosov & O'Donnell, 2019*a*). This situation is proposed to be an adaptation to contend with the high throughput of food, such as leaves, in larval Lepidoptera (Ramsay, 1976). Bases such as HCO_3_^−^ are secreted into the midgut lumen, maintaining it as an extremely alkaline environment to enable effective digestion of resistant foods such as leaves. These ions travel along the gut and are recovered by reabsorption in the hindgut, to complete a closed acid–base loop. Regions of the MpTs associated with the midgut are involved in base secretion, and MpT regions associated with the hindgut are associated with base reabsorption (in two regions, the ileac plexus and the CNC) (Fig. 1C; Kolosov & O'Donnell, 2019*a*; Moffett, 1994).

Cycling of K^+^ within the insect body is also apparently widespread, including in those without a CNC (Ramsay, 1953, 1953; Shaw, 1955; Irvine, 1969). The secretion and reabsorption of K^+^ are unlikely to be concerned with maintaining homeostasis of this ion, as secretion of K^+^ continues when there is no inward flux of K^+^, and significant reabsorption occurs even when K^+^ is in excess in the animal (Ramsay, 1953, 1953). Rather K^+^ transport is intimately coupled to other processes. It is thought to drive secretion of Cl^−^ ions into the MpTs and their subsequent reabsorption in the proximal MpT and rectum. There is also evidence that Cl^−^ may be actively reabsorbed in the rectum. The osmotic gradient generated by the transport of potassium chloride in turn drives the flux of water (see references within Halberg & Denholm, 2024). In the MpTs of Lepidoptera it has also been shown that the transport of Cl^−^ ions drives reabsorption of HCO_3_^−^ back into the haemolymph, underpinned by Cl^−^/HCO_3_^−^ exchangers (Kolosov & O'Donnell, 2020).

The CNCs of lepidopterans have also been identified as a site of K^+^, Cl^−^ and Na^+^ transport from the rectal contents into the MpTs (Ramsay, 1976; Irvine, 1969; Liao *et al*., 2000; O'Donnell & Ruiz‐Sanchez, 2015; Kolosov *et al*., 2018*b*), and the cryptonephridial arrangement may provide a more efficient means to absorb ions. The rectal contents are the primary source of these ions, in contrast to tenebrionid beetles where ions are transported into the CNC from the haemolymph. In Lepidoptera, ions from the haemolymph are additionally transported into the CNC in response to antidiuretic hormone signalling. This could generate a higher osmolarity around the rectum to allow greater water recovery and so maintain water homeostasis (Kolosov & O'Donnell, 2019*a*).

The ions secreted into the MpTs of the lepidopteran CNC could subsequently function in more proximal tubule regions, including in the ileac plexus. In response to a dietary deficiency of these ions, their transport in the tubules of the ileac plexus is radically altered, allowing them to be obtained from the haemolymph (Kolosov *et al*., 2018*b*, 2019; Kolosov & O'Donnell, 2019*b*, 2020; Kolosov, Leonard & O'Donnell, 2021; Kolosov, Piermarini & O'Donnell, 2018*a*). This is presumably to maintain other important transport functions requiring these ions, such as recycling of bases, uptake of water, and removal of nitrogenous waste or toxins (Kolosov & O'Donnell, 2019*a*). Furthermore the uptake of water from the rectal contents can be upregulated hormonally, and this increased uptake requires Na^+^, K^+^ and Cl^−^ (Audsley *et al*., 1993).

#### (c) Excretion of nitrogenous waste and toxins

The cycles of K^+^ and bases mentioned above have a further suggested role in the excretion of nitrogenous waste. This is based on evidence from *Rhodnius prolixus* (kissing bug, which lacks a CNC) and *M. sexta* (tobacco hornworm, which has a CNC). Nitrogenous waste is likely maintained in a soluble form such as urate for secretion into the MpT, and is then subsequently precipitated as uric acid upon reabsorption of KHCO_3_ into the haemolymph (Edney, 1957; Wigglesworth, 1974*a*; Moffett, 1994). The endocrine regulation of ionic cycling in order to clear metabolic waste is discussed in Section (4). Removal of other toxins by the MpTs can rely on transport of specific ions in some cases, and on general fluid flux in others (O'Donnell, 2009).

The CNC of tenebrionid beetles thus has primarily been implicated in the conservation and acquisition of water. In addition to such a water‐conservation function, the CNC of lepidopteran larvae has also been implicated in the recycling of ions, likely to underpin maintenance of pH balance, and excretion of waste compounds. It is unclear whether tenebrionid beetle CNCs also act in these additional roles. It is possible that the predominant role of the CNC differs among groups or species, however in all cases CNCs seem to function as powerful recycling systems.

### (2) CNC structure in tenebrionid beetles

#### (a) Organisation of the CNC

*Tenebrio* and *Tribolium* have an extremely similar CNC organisation (King & Denholm, 2014; Ramsay, 1964; Saini, 1964; Naseem *et al*., 2023). Their CNC incorporates the rectum (i.e. the posterior‐most part of the hindgut) excluding a short section close to the anus, and the distal regions of the six MpTs, the blind ends of which reside near the rectum's posterior end. The MpTs have a sinuous course, and in the posterior part they are highly folded and tightly packed together. Here they have a “beads on a string” appearance, created by a series of swellings along their length termed boursouflures (Fig. 1A). Their walls are thick and their lumens very narrow, with their cells having highly elaborated brush borders on their apical/luminal surface. The CNC also contains longitudinal and circular muscles which lie between the hindgut epithelium and the MpTs (Grimstone, Mullinger & Ramsay, 1968; King & Denholm, 2014; Ramsay, 1964) (Fig. 1E).

Ensheathing the complex is a tissue termed the perinephric membrane. In specific regions within the perinephric membrane small windows are present, replaced instead with a thin blister which bulges into the haemolymph. Such blisters overlie specialised MpT cells, the leptophragmata. A single leptophragma resides at the centre of the external surface of each boursouflure (Ramsay, 1964; Grimstone *et al*., 1968).

In *Tenebrio* and coccinellid beetles the perinephric membrane forms a tight seal around the complex, forming a compartment, isolated from the body cavity, known as the perinephric space. Experiments with injection of Indian ink into the perinephric space reveal it to be isolated from the haemolymph for most its length. However, it appears that the perinephric membrane may be less tightly bound at its very anterior end (Ramsay, 1964; Pradhan, 1942). It has been argued that such an unsealed sleeve could act as a valve, allowing excess fluid to escape but restricting the inflow of fluid (Ramsay, 1964).

#### (b) The perinephric membrane

The perinephric membrane is a tissue layer comprising an outer and inner sheath. A layer of basement membrane externally surrounds the outer sheath. The blisters overlying the leptophragmata are formed from a multi‐layered basement membrane, and lack cells (Grimstone *et al*., 1968). Multilayering of basement membranes has been considered an adaptation for increased mechanical strength (Sawada & Inoue, 1996). The outer sheath is a thin single cell layer, and it was suggested from electron micrographs that the outer parts of these cells are packed with microtubules (Grimstone *et al*., 1968), however fluorescence microscopy has revealed that the outer sheath is predominantly enriched in F‐actin (R. Beaven, K.V. Halberg & B. Denholm, in preparation). The inner region of the outer sheath cells contains abundant mitochondria and other organelles.

The inner sheath of the perinephric membrane is a tissue composed of many extremely thin cellular layers (as many as 40 in *Tenebrio*, sometimes <10 nm thick), forming a multilaminated structure. Grimstone *et al*. (1968) commented on its resemblance to the myelin sheaths that insulate vertebrate neurons. No similar structure had been reported in insects, however recently glia have been shown to form laminated myelin sheaths at the junction of the central and peripheral nervous system in adult *Drosophila melanogaster* (hereafter *Drosophila*) (Rey *et al*., 2023). In some of the layers of the inner sheath are regions that appear to contain many parallel microtubules. It is unclear if individual cells in this tissue have been folded or wrapped many times to generate the perinephric membrane, or if each layer represents a distinct cell (Grimstone *et al*., 1968). Nuclei have only been seen in the layer of cell bodies at the inner side of the inner sheath, and the outer part of the inner sheath has therefore been interpreted as consisting of the folded or coiled layers of these cells (Koefoed, 1971). The perinephric membrane does not extend over the leptophragmata, except for the thin blister. The ends of the multiple layers of the inner sheath insert into ridges in an electron‐dense extracellular ring around the periphery of the leptophragmata, and in some places the neighbouring MpT cells (principal cells, see Section (c)) (Grimstone *et al*., 1968).

We have gained evidence from adult *Tribolium* that the outer sheath shares its developmental origins and aspects of its differentiation with muscle, displaying striated patterns of F‐actin, α‐actinin and myosin heavy chain staining. There are no indications however that this layer is contractile, and thus the outer sheath cells may rather perform a structural role (R. Beaven & B. Denholm, unpublished observations).

#### (c) The Malpighian tubules

*Tribolium* and *Tenebrio* have six MpTs which, at their proximal end, drain into the gut at the midgut‐hindgut boundary. The proximal MpT region lies freely within the haemolymph and is termed the free tubule. The free tubules come together in a single bundle termed the common trunk, which connects with the anterior end of the CNC. From here the tubules run a sinuous course over the rectal surface, to where their blind distal ends terminate at the posterior end of the CNC (Ramsay, 1964; King & Denholm, 2014; Fig. 1A). The free MpTs are netted in muscle and undergo peristaltic contractions. These result in a back‐and‐forth motion of the fluid in the lumen extending into the MpTs of the CNC. This ultimately results in a net movement of fluid out of the CNC tubules into the free tubules (Ramsay, 1964). The free MpTs are considered the site of water reabsorption back into the haemolymph. This would need to be driven by active reabsorption of salts as the fluid in the MpT lumen exiting the CNC is hypertonic relative to the haemolymph (Machin, 1979).

The free tubules are comprised of principal cells, and a smaller number of secondary cells, regularly dispersed along their length. These two major cell types of the MpTs appear to be evolutionarily conserved, with the two types also known in *Drosophila* (King & Denholm, 2014; Denholm *et al*., 2013). A small subpopulation of MpT principal cells, seemingly unique to beetles, is under specific neuropeptide regulation. These have been referred to as “‘inverse secondary cells” (Halberg *et al*., 2015). Within the MpTs of the CNC, the highly specialised leptophragmata are found among principal cells, and have been identified as a specialised form of secondary cell (Beaven *et al*., 2024*b*). The leptophragma has a very unusual morphology, forming an extremely thin disc which separates the MpT lumen from the overlying blister, and has a cell body which hangs down into the MpT lumen (Fig. 1F). In the more anterior part of the CNC the perinephric membrane is thinner, the MpTs are less tightly folded and packed, and their walls are thinner and lumens larger. The MpTs also lack boursouflures in this region (Ramsay, 1964; Grimstone *et al*., 1968). The secondary cells of the anterior CNC are morphologically and molecularly distinct from leptophragmata (Beaven *et al*., 2024*b*).

The junctional interface of the leptophragmata and their neighbouring principal cells appears similar to that described at the interface between cells of the insect midgut epithelium, which display interdigitations, desmosomes and septate junctions (Li *et al*., 2018; Silva‐Olivares *et al*., 2003; Shanbhag & Tripathi, 2009). The midgut and MpTs are known to show commonalities in their junctional components, including presence of the same smooth septate junction proteins (Izumi *et al*., 2016; Jonusaite *et al*., 2020; Jonusaite, Donini & Kelly, 2017; Yanagihashi *et al*., 2012; Dornan *et al*., 2023), so this is likely to be a junctional interface typical for MpT cells.

#### (d) The rectum

On the luminal surface of the rectal epithelium is a cuticle. In most insects investigated, the rectal cuticle has a high permeability to water, including a generally higher permeability than the foregut cuticle (Maddrell & Gardiner, 1980). The rectal cuticle also acts as a molecular sieve and is particularly permeable to small hydrophilic molecules (Phillips *et al*., 1987). In larvae of *Tenebrio* and *Onymacris* species, absorption of water vapour does not occur around the time of ecdysis (Machin, 1975; Coutchié & Crowe, 1979). It is conceivable that this is related to an active role of the rectal cuticle in facilitating water movement. It is known that the cuticle covering the body of certain tenebrionid beetles of the Namib desert possesses highly specialised structures which allow them to collect water droplets from fog (Parker & Lawrence, 2001), and it is possible that the cuticle lining the rectal lumen also has structural properties important for its interaction with water. Alternatively, interruption to the water absorption capabilities of the complex during moulting may result from endocrinological regulation of the CNC during ecdysis, or inhibition of water movement by the newly forming cuticle.

### (3) CNC physiology

#### (a) Osmotic gradients within the CNC, and the movement of water

In *Tenebrio*, water is absorbed from the rectal lumen into the perinephric space surrounding the cryptonephric MpTs, and then into the MpT lumen. From here fluid flows into the free MpT regions, from where it can be reabsorbed into the haemolymph, driven by the active reabsorption of salts. There is an increase in osmolarity in each compartment moving from the rectal lumen to the lumen of the cryptonephric MpTs, which accounts for the movement of water, and appears to be driven by the accumulation of potassium chloride in the perinephric space and MpT lumen (Fig. 1D).

The movement of water may occur *via* a paracellular route, through the septate junctions between cells. There is evidence from the MpTs of diverse insect species, that this is a regulated route for the movement of water and ions (Hernández, González & Whittembury, 1995; Nicholls, 1983; Beyenbach, 2003; Kolosov *et al*., 2019). However the main route by which water crosses the MpT epithelium is likely to be transcellular, *via* plasma membrane‐localised channels such as aquaporins (O'Donnell & Maddrell, 1983; Cabrero *et al*., 2020).

The osmolarity in the perinephric tubule lumen is higher towards the posterior end of the complex, and at the most posterior it is close to the value required to achieve water absorption from air with a relative humidity of ~90% (Ramsay, 1964; Machin, 1979). The role of the perinephric membrane is apparently to insulate the whole complex from the free exchange of water and ions with the haemolymph. There is evidence that it is impermeable to the movement of water (Ramsay, 1964), particularly in the posterior region where its inner sheath is more substantial, as compared to the anterior region (Grimstone *et al*., 1968).

The leptophragmata are thought to be impermeable to water, so the concentration of potassium chloride can increase within the cryptonephric MpTs and the perinephric space (Grimstone *et al*., 1968). This property of the leptophragmata of being seemingly permeable to Cl^−^ but impermeable to water is considered highly unusual (Koefoed, 1971). In Diptera and Lepidoptera, the secondary cells act as sites of Cl^−^ transport (Cabrero *et al*., 2014; O'Connor & Beyenbach, 2001; Kolosov & O'Donnell, 2020), as well as water movement (Cabrero *et al*., 2020; Kaufmann *et al*., 2005). If the leptophragmata are modified secondary cells (see Section (3)), an important modification may have been the loss of this water movement through leptophragmata. However there is also evidence that water flux may not occur in the secondary cells of the *Tribolium* free tubule either (Cabrero *et al*., 2020). Further, Drip, an aquaporin water channel, which localises to the apical surface of secondary cells in diverse insects (Cabrero *et al*., 2020; Kaufmann *et al*., 2005), does not show clear localisation to any site in the MpT in the beetle *Anomala cuprea* (Nagae *et al*., 2013). Together these data suggest the ability to transport water may have been generally lost from secondary cells within Coleoptera.

#### (b) Potassium chloride transport in the CNC

Potassium chloride appears to be the key driver of water transport by the CNC in both *Tenebrio* and *Onymacris* species (Machin & O'Donnell, 1991). Evidence from *Onymacris* shows an active transport of K^+^ from the perinephric space into the MpT lumen, and a mainly passive movement of Cl^−^ (Machin & O'Donnell, 1991). There is also evidence that Na^+^ is actively accumulated in the CNC in *Tenebrio* and *Onymacris*, although it does not appear to be a major contributor to the osmolarity of the fluid within the tubules, nor to the ability of the complex to transport water (Tupy & Machin, 1985; Machin & O'Donnell, 1991; O'Donnell & Machin, 1991).

K^+^ can be secreted into the MpTs of the CNC from the haemolymph, showing that there is a transport route between these two compartments, but there is high resistance to the movement of water along this route (Ramsay, 1964; Tupy & Machin, 1985). When moving from the haemolymph into the CNC, K^+^ is secreted against a concentration gradient (Grimstone *et al*., 1968). The perinephric membrane does not seem to be involved in movement of K^+^, as its cells lack the subcellular structures associated with active ion transport (Ramsay, 1976; Kolosov & O'Donnell, 2019*a*).

The leptophragmata were initially identified as sites of free passage of Cl^−^. High Cl^−^ concentration in the leptophragmata was revealed by incubation with sliver nitrate, which reacts to produce silver chloride which, in turn, forms atomic silver in the presence of light, visible as a dark staining (Ramsay, 1964; Lison, 1937; Grimstone *et al*., 1968). Cl^−^ ions are thought to move passively into the MpT lumen, driven by the accumulation of positively charged ions such as K^+^ in this compartment (Machin & O'Donnell, 1991; O'Donnell & Machin, 1991). The route by which K^+^ moves from the haemolymph into the MpT lumen has remained less clear. It has been considered that the leptophragmata are also a plausible route for the transport of K^+^ (Grimstone *et al*., 1968; Ramsay, 1964), and measurements of high K^+^ concentration in the leptophragmata have been reported (Marshall & Wright, 1972), although measurement of K^+^ concentration in different compartments suggested that this ion moves passively from the haemolymph into the perinephric space, from where it is actively pumped into the MpT lumen (Machin & O'Donnell, 1991; O'Donnell & Machin, 1991).

It was recently shown that an ion antiporter, Na^+^, K^+^/H^+^ antiporter 1 (NHA1), is expressed in the leptophragmata of *Tribolium* and *Tenebrio* (Naseem *et al*., 2023). NHA1 expression is upregulated in response to desiccating conditions, and this antiporter was found to be important for maintaining water balance. NHA1 appears to be the sole NHA member in *Tribolium*, whilst other insects typically possess two paralogues. NHAs are from the cation/proton antiporter‐2 (CPA2) subfamily of proteins, which typically act as Na^+^/H^+^ antiporters, but can transport other ions. Electrophysiological characterization in *Xenopus* oocytes suggests that NHA1 is a K^+^/H^+^ antiporter, and could therefore mediate transport of K^+^ from the haemolymph into the CNC, *via* the leptophragmata (Naseem *et al*., 2023; Fig. 1E,F).

#### (c) Energisation and metabolism of the CNC

NHA1 transport of K^+^ would in turn rely on the active transport of protons. Vacuolar ATPases (V‐ATPases) are protein complexes known to transport protons across insect epithelia, energised by the hydrolysis of ATP (Wieczorek *et al*., 1999). V‐ATPase protein components have been found to localise to the apical brush border of principal cells within MpTs of the CNC (Naseem *et al*., 2023), a region also rich in mitochondria (Grimstone *et al*., 1968). It has therefore been hypothesised that V‐ATPase establishes a proton gradient to drive K^+^ transport from the haemolymph into the tubule lumen through NHA1 in the leptophragmata (Naseem *et al*., 2023; Fig. 1F).

The energy requirements for ion transport of the CNC would create a high metabolic load, and demand for oxygen. In the posterior region of the CNC, the small tracheolar cells which form the end of the tracheal network are sandwiched between the inner and outer perinephric membranes (Grimstone *et al*., 1968). Tracheole cells have also been identified in both the inner and outer sheath of the perinephric membrane (Koefoed, 1971). This is likely to be due to the oxygen requirements of the CNC. Trachea also extend into the perinephric membrane of the CNC of the New Zealand glow worm *Arachnocampa luminosa* (Wheeler & Williams, 1915), and the ant lion *Acanthaclisis* spp. (Loziński, 1911; Poll, 1936), as well as associating with the CNC of the fire ant *Solenopsis saevissima* (Arab & Caetano, 2002). Dense tracheation is seen in the rectal pads of many species which do not have CNCs, thought to serve the metabolic costs that underly the water reabsorption function in this organ (Chapman, 1998*a*); it is likely the dense tracheation in CNCs serves the same function.

### (4) Endocrinological control of CNC activity in tenebrionid beetles

Control of insect osmoregulation typically is exerted through endocrine regulation, to enable water and solute balance to be homeostatically maintained in response to a changing external environment and ingested food. The picture that has emerged from beetles is of major evolutionary changes to their endocrine signalling. Genomic studies show the absence of several genes encoding diuretic neuropeptides. For example kinin, which activates Cl^−^ transport in the secondary MpT cells of dipterans (Terhzaz *et al*., 1999; Radford, Davies & Dow, 2002), is absent in most beetles, only being found within Adephaga (Li *et al*., 2008; Pandit *et al*., 2019; Hauser *et al*., 2008). A further difference is the presence in beetle genomes of the gene encoding vasopressin which is absent in many other insects. Vasopressin in beetles stimulates MpT secretion (Li *et al*., 2008; Pandit *et al*., 2019; Hauser *et al*., 2008; Aikins *et al*., 2008). The ways in which endocrine pathways regulate MpT physiology also seem to have undergone major evolutionary changes in beetles. For example we know in *Drosophila* that capa and diuretic hormone 31 (DH_31_) signal to the MpT principal cell population to upregulate cation and fluid secretion (Terhzaz *et al*., 2012; Coast *et al*., 2001), however this signalling is restricted to a morphologically distinct subset of principal cells (named inverse secondary cells) in beetle species where it has been studied (Halberg *et al*., 2015).

In dipterans, the corticotropin releasing factor (CRF) member DH_44_ signals *via* the DH_44_‐R2 receptor to stimulate secretion, again acting in the principal cells (Jagge & Pietrantonio, 2008; Hector *et al*., 2009; Cabrero *et al*., 2002; Cannell *et al*., 2016). A related pathway was identified in *Tenebrio*, mediated by the neuropeptides, DH_37_ and DH_47_ (Wiehart *et al*., 2002). Although in beetles from the suborder Adephaga, this pathway also acts upon the principal cells, in the suborder Polyphaga (including in *Tribolium*) it acts *via* the secondary cells of the free tubule regions, which express urinate receptor (Urn8R), a receptor related to dipteran DH_44_‐R2. DH_37_ and DH_47_ are expressed in specific neurons within the brain, and function to stimulate secretion within the free MpT (Koyama *et al*., 2021) (Fig. 1G).

Far less is known about the endocrine control of the CNC in tenebrionid beetles. Urn8R was found to be expressed specifically within the secondary cells of the anterior portion of the CNC, similar to in the free tubule, but was not detected in the leptophragmata which reside in the more posterior CNC. The transcription factor Dachshund is expressed in the distal MpT including the leptophragmata (which are specialised secondary cells, see Section (3)) but not in the secondary cells of the anterior CNC or the free tubule. Dachshund functions to repress Urn8R expression in the leptophragmata (Fig. 1F), and future investigations could determine whether this is important to prevent an inappropriate osmoregulatory response to DH_37/47_ signalling (Beaven *et al*., 2024*b*). We speculate there could be good reasons why secretion in free *versus* cryptonephric tubules have evolved separate hormonal regulation systems in beetle groups possessing CNCs. If secretion activity was stimulated throughout the entire MpT, this would result in the cryptonephridial tubules removing more water from the rectum, so countering diuresis driven by increased secretion in the free tubule, preventing the animal from expelling excess water.

It was previously shown that diuretic hormone treatment of *Tenebrio*, and of the desert tenebrionids *Physadesmia globose* and *Onymacris* species, causes elevated secretion in the free MpT. It was speculated, however, that this may generate an increased cycling of fluid, rather than whole‐animal diuresis, and could function in clearance of metabolic waste (Wiehart *et al*., 2002; Nicolson, 1992; Nicolson, 1991; Nicolson & Hanrahan, 1986). On this basis Nicolson suggested that “clearance hormone” may be a more appropriate term than diuretic hormone in these cases (Nicolson, 1991). This notion fits with findings from Lepidoptera, where diuretic hormone stimulates fluid transport in both the free MpTs and the CNC. This simultaneous diuretic and anti‐diuretic effect is thought to increase fluid cycling rather than having a truly diuretic effect at the whole‐animal level (Audsley *et al*., 1993). However in *Tribolium*, activation of the DH_37/47_–Urn8R pathway has been found to stimulate whole‐animal diuresis (Koyama *et al*., 2021), likely enabled by restricting regulation to cells only in the free tubule and anterior CNC.

## III. DEVELOPMENT AND EVOLUTION OF THE TENEBRIONID BEETLE CNC

Novel structures or organs, which differ dramatically from their forerunners and arise by discrete evolutionary events rather than through gradual change, are considered key drivers of the emergence of new species and the exploitation of new niches. The problem of how they arise is a longstanding central question at the interface of development and evolution (DiFrisco, Wagner & Love, 2023; Almudí & Pascual‐Anaya, 2019; Moczek, 2023; Brigandt & Love, 2012; Mayr, 1960). Parts of the CNC appear to have evolved from ancestral organs and tissues including the rectum, and the MpTs, as well as a seemingly novel tissue, the perinephric membrane. Together these took a new configuration to create a new organ system with integrated function. The CNC therefore provides an example of an evolutionary novelty that has arisen through fusion, which was previously proposed as a route by which novel features arise (Wagner, 2014; Oakley, 2017; Brückner *et al*., 2021; Almudí & Pascual‐Anaya, 2019). The perinephric membrane appears to be a novel tissue. It may be derived from an ancestral mesodermal tissue such as the visceral muscle, but its structure does not resemble any known insect mesodermal tissue. It may have recruited molecular pathways from other cell types such as glia, for example intracellular signalling pathways, their downstream cytoskeletal and membrane regulators, and their subcellular structures. Glia have been shown to form myelin‐like structures in the nervous system of *Drosophila* (Rey *et al*., 2023). Developmental studies are shedding light on the means by which novelties have arisen (Prud'homme *et al*., 2011; Hu *et al*., 2018; Ohde, Yaginuma & Niimi, 2013; Moczek, 2023), and studying embryonic development of the CNC has the potential to shed further light on this question.

Nothing was known about CNC formation in any insect until recent studies, which are beginning to unravel the developmental processes underlying CNC formation in *Tribolium* embryos. The constituent parts of the CNC in *Tribolium* are already assembled in their mature configurations in newly eclosed larvae, and although they undergo significant growth by adulthood, they do not appear to undergo major rearrangements, including during metamorphosis (King & Denholm, 2014; R. Beaven & B. Denholm, unpublished observations). Insights into the processes and molecular players mediating CNC development in *Tribolium* are outlined below to provide a starting point for future comparative studies with species lacking a CNC, and species which have independently evolved a CNC, in order to understand the evolution of these novel organ systems.

### (1) Development of the MpTs

**Fig. 2:**
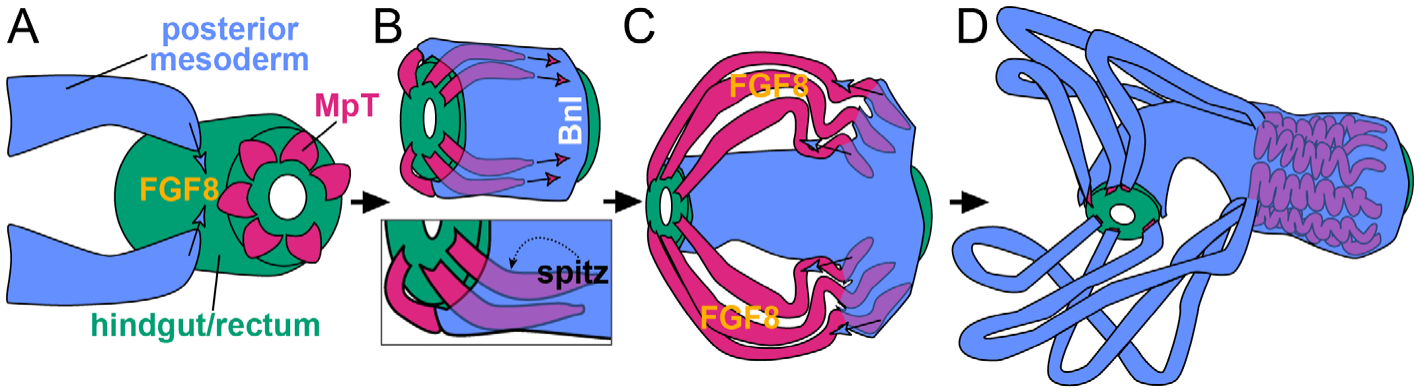
Embryonic development of the cryptonephridial complex (CNC) in *Tribolium*. (A) Six Malpighian tubules (MpTs) bud out from the end of the developing hindgut/rectum. The hindgut/rectum expresses the fibroblast growth factor 8 (FGF8) ligand. In *Tribolium*, a single FGF receptor (FGFR) gene is expressed as distinct isoforms, which receive the different FGF ligands. The FGFR isoform for FGF8 is expressed in a posterior population of mesoderm. FGF8 signalling recruits the posterior mesoderm onto the hindgut/rectum. (B) A second FGF ligand, Branchless (Bnl) becomes expressed in the region of posterior mesoderm now surrounding the posterior end of the developing rectum. Tip cells, which guide the growing MpTs, express the FGFR isoform for Bnl. This pathway guides the MpTs to grow into the posterior mesoderm, closely surrounding the hindgut/rectum. The epidermal growth factor (EGF) ligand spitz signals from the distal end of the MpT, and likely drives and orients MpT elongation. (C) FGF8 continues to be expressed in the hindgut/rectum, but also switches on in the MpTs, and is likely responsible for some of the posterior mesoderm migrating over the MpT surface. (D) The posterior mesoderm coats the MpTs and hindgut, and will differentiate into the perinephric membrane, and muscle cells, for example those that form a mesh around the free MpTs. The distal ends of the MpTs have established their countercurrent organisation with the rectum, and become convoluted, so entirely surrounding the rectum.

The molecular and cellular mechanisms of MpT development have been extensively studied in *Drosophila* which lacks a CNC. This provides a useful starting point for considering development of the MpTs of *Tribolium*, as well as a useful comparison to understand the evolutionary changes in developmental processes that have given rise to the CNC. In many respects MpT development appears to be similar in the two species (Beaven *et al*., 2024*b*; King & Denholm, 2014; Jung *et al*., 2005; Denholm, 2013). In *Tribolium*, the MpT primordia at the future anterior end of the hindgut, begin expressing the transcription factor Cut, and this persists throughout their development. Distinct tubules bud out (six in *Tribolium*, four in *Drosophila*; Fig. 2A) and undergo cell proliferation. The distal tubule end is a source of the epidermal growth factor (EGF) ligand, Spitz, in both species. In *Tribolium*, this is achieved by restriction of Spitz expression to the tip cell, and, at a lower level, to a small cluster of distal cells. In *Drosophila* it is achieved by specific cleavage of Spitz in the tip cell and neighbouring sibling cell. A gradient of EGF signalling activity occurs in both species (King & Denholm, 2014; Saxena *et al*., 2014). In *Drosophila* this EGF signalling establishes planar cell polarity in the MpT cells, to drive their intercalation (Saxena *et al*., 2014), and EGF signalling also drives cell proliferation in the distal MpT (Sudarsan *et al*., 2002). These two processes underly elongation of the developing MpTs. Similar pathways are likely to be used in *Tribolium*, in which oriented cell divisions align with the proximo‐distal axis of the MpT, so driving its elongation (King & Denholm, 2014). Planar cell polarity is known to orient cell division in other contexts (Gho & Schweisguth, 1998; Saburi *et al*., 2008).

All six tubules orient their growth to surround the developing rectum closely, and end up with their tip cells close to the posterior end of the rectum. This differs from the positions of the MpTs in *Drosophila*. In *Drosophila* the length of the MpTs remain free from other organs, with the distal tip cells of the anterior MpT pair anchoring to specific alary muscles (Weavers & Skaer, 2013), and the posterior pair tethering to specific neurons adjacent to the hindgut (Hoch *et al*., 1994). In *Tribolium* the more proximal section of each MpT continues to grow, forming a free loop which extends towards the anterior of the animal. The distal tubule buckles to form a regular concertinaed morphology. This concertinaing travels in a wave from the distal tubule end, in a proximal direction, and the tubules continue to elongate. These morphological changes mean that by late embryogenesis the tubules come to have a tightly packed wave like course, which entirely surrounds the rectum, separated from the rectal epithelium by a layer of visceral muscle (King & Denholm, 2014).

Each tip cell extends an actin‐rich structure ahead of the advancing tubule (King & Denholm, 2014), which is likely involved in pathfinding of the growing tubule in response to guidance cues, and may also be involved in recognition and adhesion to target cells. A role of the tip cell in anchorage to specific target cells has been shown in *Drosophila* (Weavers & Skaer, 2013). In *Tribolium*, the fibroblast growth factor (FGF) ligand Branchless (Bnl) is produced in the most posterior region of a mesodermal cell population which gives rise to the perinephric membrane, close to the posterior end of the rectum (Fig. 2B, and see Section (2)). Bnl signals to the tip cells, and disrupting this pathway leads to loss of the actin‐rich tip cell structures and misrouting of the MpTs (R. Beaven, K.V. Halberg & B. Denholm, in preparation). These phenotypes are similar to those seen in the tip cells of the *Drosophila* trachea following perturbation of the Bnl pathway (Sutherland, Samakovlis & Krasnow, 1996; Ribeiro, Ebner & Affolter, 2002). The guidance of *Tribolium* tubule tip cells by Bnl during development establishes the close association of the MpTs with the rectum, and the countercurrent orientation of the MpTs in relation to the rectum. The different final positions of the MpT tip cells seen between *Tribolium* and *Drosophila* may result from evolutionary recruitment or modification of Bnl signalling to bring about the reorganisation of the insect internal anatomy from an ancestral non‐cryptonephridial state (more akin to *Drosophila*), to generate the derived cryptonephridial organisation seen in many beetles.

### (2) Development of the perinephric membrane

Development of the perinephric membrane, and the cell types it is derived from has remained obscure until recent investigations. The mesodermal fat body (Riechmann *et al*., 1998) associates with the free MpTs in spiders (which lack a CNC) and ant lion larvae (which have a CNC) (Poll, 1936; Butt & Taylor, 1986). The fat body is therefore one possible evolutionary origin of the perinephric membrane. Indeed in the New Zealand glow worm, which has a CNC with a perinephric membrane, its perinephric membrane is thought to be derived from long sections of fat body which extend from its oesophageal valve (Green, 1980).

In *Tribolium*, there is also evidence that the perinephric membrane is mesodermal, although it seems more similar to muscle than fat body. A population of mesodermal cells in the posterior region of the embryo migrates to the rectum and develops into the perinephric membrane. This population expresses the transcription factors twist (R. Beaven, K.V. Halberg & B. Denholm, in preparation), which is required to specify mesodermal fate in *Tribolium* as it is in *Drosophila* (Sommer & Tautz, 1994; Handel *et al*., 2005; Stappert *et al*., 2016), and sex‐determining region Y‐Box D (SoxD) which is involved in mesoderm and nervous system differentiation in panarthropods (Janssen *et al*., 2018; R. Beaven, K.V. Halberg & B. Denholm, in preparation). These perinephric membrane precursor cells are distinct from the visceral muscle precursors, and unlike these do not express Cut (King & Denholm, 2014; R. Beaven, K.V. Halberg & B. Denholm, in preparation). It appears that this posterior mesoderm gives rise to both the inner and outer sheath of the perinephric membrane. We have observed that the outer layer takes on muscle‐like characteristics, with a striated pattern of F‐actin, α‐actinin and myosin heavy chain staining (R. Beaven & B. Denholm, unpublished observations), consistent with a mesodermal origin. This mesodermal cell population migrates onto the developing hindgut during embryogenesis, and the MpTs extend into this tissue, under the guidance of Bnl (see Section (1)). By late embryogenesis the posterior mesoderm migrates to cover the complex and also migrates to surround the free tubule, and is likely also to give rise to the mesh of muscle which forms around the mature free tubule (Ramsay, 1964; King & Denholm, 2014; R. Beaven, K.V. Halberg & B. Denholm, in preparation).

Signalling by a further FGF ligand, named FGF8, appears to recruit the posterior mesoderm cells, with FGF8 initially expressed in the developing hindgut alone (Fig. 2A), and subsequently in the hindgut and MpTs (Fig. 2C). Two distinct FGF signalling pathways (FGF8 and Bnl) therefore orchestrate a complex series of interactions between the hindgut, developing perinephric membrane and MpTs to assemble the CNC (R. Beaven, K.V. Halberg & B. Denholm, in preparation; Fig. 2).

FGF signalling between the MpTs and perinephric membrane persists through larval and pupal stages into adulthood. It may also function in the final differentiation and maturation of the perinephric membrane, particularly the inner sheath, with its unusual, laminated structure, although this has not yet been demonstrated directly. Curiously, FGF8 signalling drives differentiation and enwrapment of *Drosophila* neurons by glia in the eye and olfactory system (Shishido *et al*., 1997; Franzdóttir *et al*., 2009; Avet‐Rochex *et al*., 2012; Wu *et al*., 2017). It is possible that the FGF8 pathway, and its downstream effectors, have been co‐opted from glia during evolution of the perinephric membrane to initiate their enwrapment of the CNC. Within the multiple laminations of the inner sheath are pockets which contain many microtubules, possibly in parallel bundles (Grimstone *et al*., 1968). In vertebrate oligodendrocytes it has been found that bundles of microtubules nucleated from Golgi outposts function in the outgrowth of processes and elaboration of the myelin sheath (Fu *et al*., 2019). The microtubules seen in the inner perinephric membrane may therefore be involved in cell remodelling that generates its laminated structure.

### (3) Development of the leptophragmata

In *Drosophila*, secondary cells originate from the mesodermal mesenchyme (Denholm *et al*., 2003). The transcription factor Teashirt, and its paralogue Tiptop, define secondary cell identity. Tiptop also marks secondary cells in the cricket *Gryllus bimaculatus* (Denholm *et al*., 2013), suggesting wide evolutionary conservation among insects. *Tribolium* possesses one Teashirt/Tiptop orthologue which is more similar to Tiptop (Shippy *et al*., 2008). *Tribolium* Tiptop is transiently expressed in the developing distal tubule (which is also seen in *Drosophila*), and later in embryogenesis becomes expressed only in a subset of cells scattered along the tubule length in both species (Denholm *et al*., 2013; King & Denholm, 2014). These scattered Tiptop‐expressing cells appear to be secondary cells, as in adult tubules they have smaller nuclei (Denholm *et al*., 2003, 2013), and represent roughly 20% of the tubule cells (Cabrero *et al*., 2004; King & Denholm, 2014; Satmary & Bradley, 1984).

Tiptop is also expressed in the leptophragmata cells of the CNC, suggesting that they are modified secondary cells. This would also be consistent with the transport of Cl^−^ ions through the leptophragmata (Ramsay, 1964; O'Donnell & Machin, 1991; Lison, 1937; Beaven *et al*., 2024*b*; Naseem *et al*., 2023), as secondary cells are the route of Cl^−^ transport in *Drosophila* (Denholm *et al*., 2013; Cabrero *et al*., 2014; O'Donnell *et al*., 1998). Tiptop appears to function in specifying and maintaining leptophragmata identity, with its knockdown resulting in abnormal cellular morphology, loss of expression of the NHA1 antiporter (Naseem *et al*., 2023), and loss of Cl^−^ accumulation (Beaven *et al*., 2024*b*).

The leptophragmata are highly specialised cells which appear markedly different from secondary cells in the free tubule, suggesting other factors must be specifying this unique identity. The transcription factor Dachshund is expressed in the distal MpT of *Drosophila* and *Tribolium* MpTs, in both principal and secondary cells (Beaven & Denholm, 2022; Prpic *et al*., 2001; Beaven *et al*., 2024*b*). In *Tribolium*, Dachshund is highly expressed in the leptophragmata, but not in secondary cells of the anterior CNC or the free tubule. Depletion of Dachshund results in morphologically abnormal leptophragmata, and remarkably the Urn8R hormone receptor, which is expressed in a subset of secondary cells but normally excluded from leptophragmata (Koyama *et al*., 2021), becomes expressed in leptophragmata following Dachshund depletion (Beaven *et al*., 2024*b*). Therefore leptophragmata appear to have evolved from a unique population of distal secondary cells present in the ancestral insect tubule, and require Dachshund to distinguish them from this wider Tiptop‐expressing secondary cell population.

## IV. ECOLOGY AND EVOLUTION OF THE CNC

### (1) Phylogenetic distribution of CNCs

The MpTs have become intimately associated with the rectum multiple times during arthropod evolution. This includes more rudimentary forms of rectal complex in which the MpTs are bound to the surface of the rectum, and more advanced rectal complexes, that is true CNCs, in which the complex is also insulated from the haemolymph by an ensheathing perinephric membrane. Considering both these classes of rectal complex, such arrangements appear to have evolved independently at least nine times in arthropods, primarily within holometabolous insects (in Coleoptera, Lepidoptera, Diptera, Hymenoptera and Neuroptera). In this section, insights into different rectal complexes are discussed, which in most cases are limited to structural information. The occurrence and form of arthropod rectal complexes are explored to shed light on their likely ecological roles and evolution.

#### (a) Occurrence in Coleoptera

**Fig. 3:**
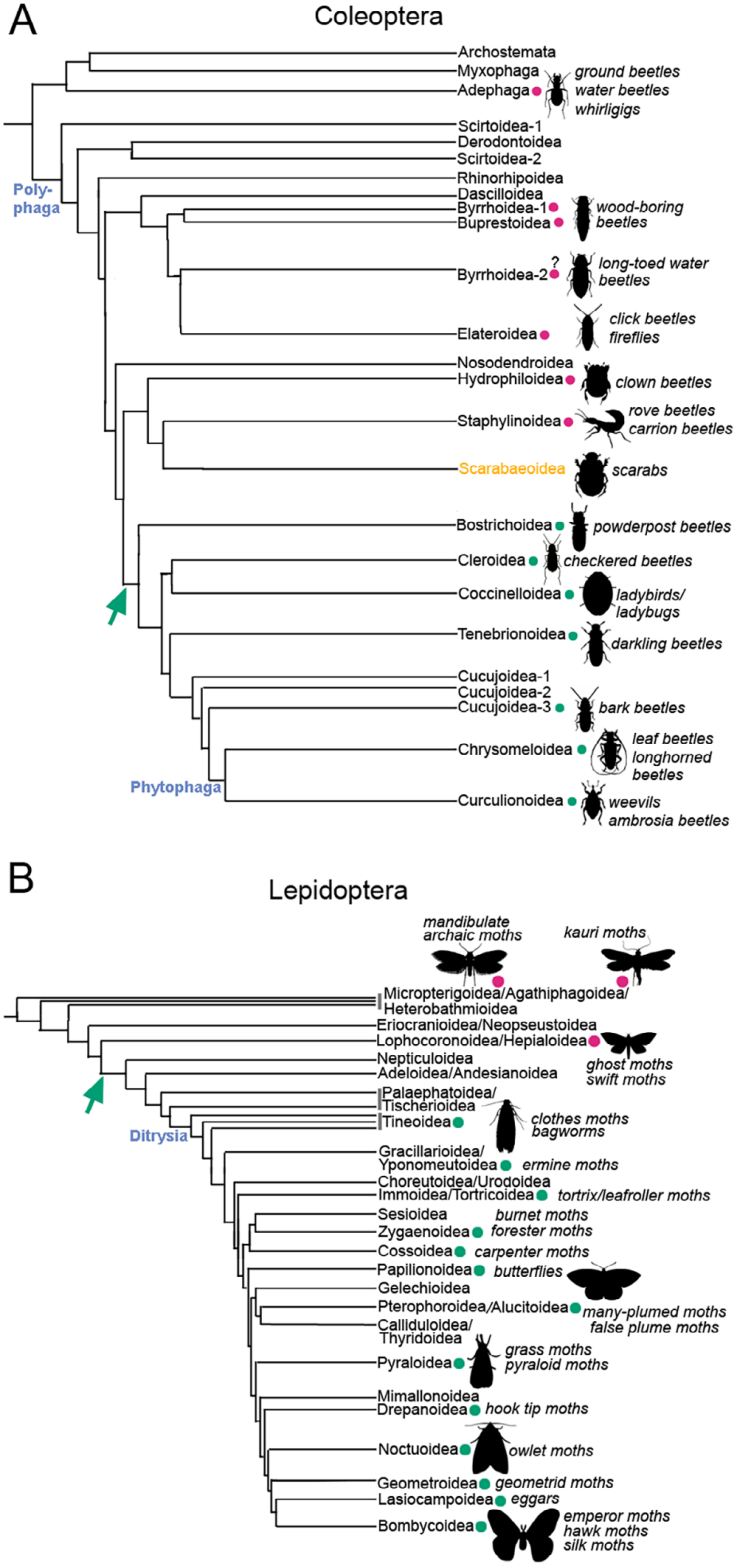
Evolutionary occurrence of the cryptonephridial complex (CNC)/rectal complex in Coleoptera and Lepidoptera. (A) Phylogenetic tree of Coleoptera, showing reports of CNC or rectal complex presence (green circles) or absence (magenta circles). The earliest likely origin of the CNC/rectal complex is indicated by the green arrow. The tree is drawn according to McKenna *et al*. (2019) and represents a ~300‐million‐year evolutionary period. Its presence in the indicated groups means that a CNC/rectal complex is likely to occur in ~190,000 known beetle species, not counting Scarabaeoidea (based on species numbers in Hunt *et al*., 2007). A rectal complex has likely evolved independently in Scarabaeoidea (light orange) (Beaven *et al*., 2024*a*). The suborder Polyphaga and the clade Phytophaga are indicated. (B) Phylogenetic tree of Lepidoptera, annotated as in A. The tree is drawn according to Kawahara *et al*. (2019) and represents a ~300‐million‐year evolutionary period. CNC/rectal complex presence in the indicated groups means that it is likely to occur in ~160,000 known species of Lepidoptera (based on species numbers in Regier *et al*., 2009). The clade Ditrysia is indicated. Further details of species and variations in the observed CNC/rectal complex structures are provided in Table S1.

As explored in the previous sections, beetles of the superfamily Tenebrionoidea have long been known to possess a CNC (Fig. 1A). CNCs are also found in the superfamilies Bostrichoidea (which includes powderpost and carpet beetles), Chrysomeloidea (which includes longhorned and leaf beetles), Cleroidea (which includes chequered beetles), Cucujoidea (which includes bark beetles), Coccinelloidea (ladybirds/ladybugs) and Curculionoidea (which includes weevils and ambrosia beetles) (Fig. 3A, see online Supporting Information, Table S1). It is likely that the CNCs of these beetle groups have a single evolutionary origin, in the common ancestor of Cucujiformia and Bostrichoidea. A CNC appears to be lacking in beetles of the suborder Adephaga (which includes ground and diving beetles), and the superfamilies Staphylinoidea (which includes rove and carrion beetles) and Elateroidea (which includes click beetles and fireflies) (Fig. 3A, Table S1), although reports from these groups remain sparse.

There is some uncertainty about beetle families falling within Byrrhoidea‐2 (see Fig. 3A). The earliest report of adult *Dryops* suggested that its MpTs are free from the rectum (Dufour, 1834). However in a later report by Hinton (1939), whilst the larval MpTs were found to have no CNC, it was suggested that adults of *Lutrochus*, as well as members of Dryopidae (long‐toed water beetles) including *Dryops* and *Helichus* do have MpTs bound to the rectum in a CNC. Adults of other families within Byrrhoidea‐2 and Byrrhoidea‐1 (Fig. 3A) were reported not to have CNCs (Hinton, 1939; Table S1). Saini's (1964) report of *Dryops* contradicts that of Hinton (1939) by describing the MpTs as emanating from the gut from where they run anteriorly over the gut surface, being bound to it by a thin membrane in two regions. From the more anterior of these bound regions, the MpTs are free within the haemolymph, looping anteriorly before running in a posterior direction so that their distal ends are close to the rectum. The MpTs are described as emanating from insertion points within the hindgut (Saini, 1964). This would seem highly unusual, as MpTs seem invariably to develop at the midgut–hindgut boundary, although it may be that a morphologically distinct posterior midgut region was mistakenly identified as the anterior hindgut. It would be useful for the gut and MpT organisation of this family to be revisited, as it could have an important bearing on the evolutionary distribution of CNCs in Coleoptera.

There are indications that rectal complexes have evolved independently within Scarabaeoidea (scarab beetles), possibly more than once (Beaven *et al*., 2024*a*; Table S1). Curiously rectal complexes in Scarabaeoidea can be larval‐ or adult‐stage specific. In the case of the subfamily Cetoniinae (flower chafers), a rectal complex is present in larvae, incorporated into the hindgut fermentation chamber, which is a large portion of the hindgut harbouring symbiotic bacteria. As in other rectal complexes, sinuous MpTs encircle the rectum, but in this case the complex is entirely enclosed by infoldings of the fermentation chamber (Beaven *et al*., 2024*a*). The function of this unusual structure remains unknown. Conversely the rectal complex reported in adults of the common cockchafer *Melolontha melolontha* (tribe Melolonthini) (Lison, 1938; Saini, 1964), is not present in the larval stage (Beaven *et al*., 2024*a*). These findings are consistent with the major remodelling of the gut that occurs during metamorphosis in Scarabaeoidea, likely to underpin changes in diet between larval and adult life (Chiang & Shelomi, 2023; Nardi *et al*., 2006). In *M. melolontha* adults, the MpTs associated with the rectum are enveloped in a very fine cellular layer (Saini, 1964). This could represent a simple form of perinephric membrane, however our findings suggest it is not a continuous sheet, so is unlikely to act as an insulating barrier (Beaven *et al*., 2024*a*). The seeming absence of rectal complexes in Staphylinoidea (Beaven *et al*., 2024*a*; Table S1), which is sister to Scarabaeoidea (Fig. 3A) provides one line of evidence for independent evolution of rectal complexes within Scarabaeoidea. So too does the apparent lack of rectal complexes in the most basal groups of Scarabaeoidea which have been surveyed, such as Lucanidae (stag beetles) (Beaven *et al*., 2024*a*; Table S1). Structural differences in the rectal complexes of Scarabaeoidea, compared to the CNCs of Cucujiformia and Bostrichoidea, would also be in line with their independent evolution. As the rectal complex of *M. melolontha* apparently develops during pupal metamorphosis, it may be generated by distinct developmental mechanisms, which could also indicate independent evolution. Systematic characterisation of the morphology of alimentary canals and MpTs across Scarabaeoidea and the related superfamilies Staphylinoidea and Hydrophiloidea (water scavenger beetles and clown beetles), in both larval and adult stages, could shed more light on rectal complex evolution within Scarabaeoidea.

#### (b) Occurrence in Lepidoptera

In Lepidoptera it appears that the CNC evolved once, and is found in caterpillars of most butterflies and moths within the clade Ditrysia, which are by far the most species‐rich group (Figs 1C and 3B, Table S1). In Lepidoptera the CNC condition is absent in the adult stage. The MpTs of the CNC are generally folded upon themselves, resulting in two layers of MpTs, with significantly different organisations of the outer MpT layer among different groups (Ishimori, 1924). CNCs have not been observed in the more basal and species‐poor groups; the suborder Zeugloptera (containing the single superfamily Micropterigoidea, the mandibulate archaic moths), suborder Aglossata (containing the superfamily Agathiphagoidea, the kauri moths), and infraorder Exoporia (which includes the superfamily Hepialoidea, the ghost moths and swift moths) (Fig. 3B, Table S1). It may be that the CNC contributed to the remarkable radiation of Ditrysia (Kawahara *et al*., 2019; Regier *et al*., 2009).

#### (c) Occurrence in Diptera

**Fig. 4:**
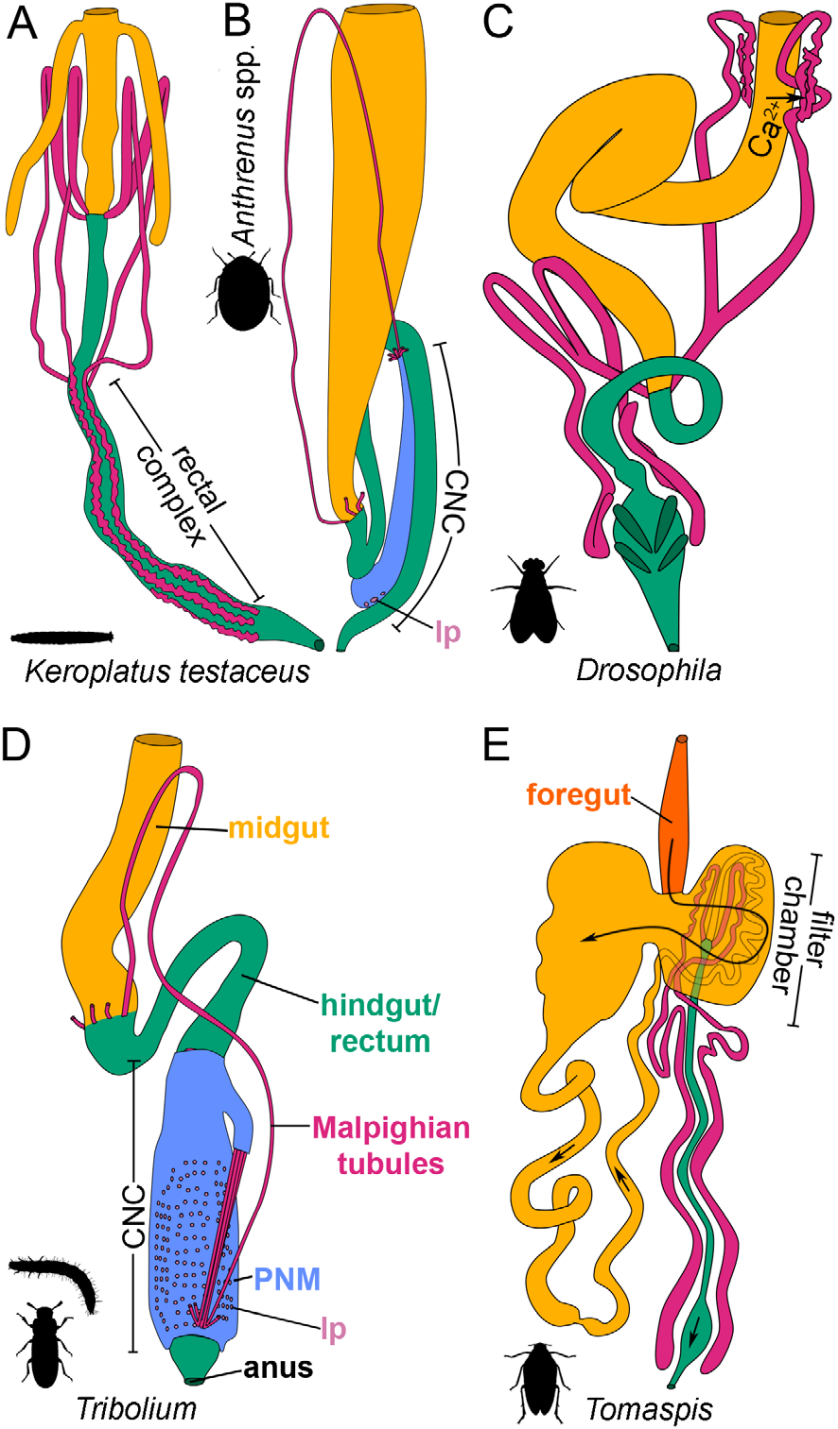
Architectures of insect digestive/renal organs. (A) Organisation seen in larval *Keroplatus testaceus* (modified from Stammer, 1932), a dipteran with a rectal complex. (B) Organisation seen in adults of *Anthrenus* species (Saini, 1964; R. Beaven & B. Denholm, unpublished observations), with a laterally displaced cryptonephridial complex (CNC). Note that the cryptonephridial Malpighian tubules (MpTs) are obscured by the perinephric membrane (PNM). A similar organisation exists in larvae (Mobüsz, 1897). lp, leptophragma. (C) Organisation seen in *Drosophila* adults, a species without a rectal complex. Calcium transport from the midgut contents to the distal regions of the anterior MpTs is indicated. Approximately equivalent positions are also seen in larvae. (D) Organisation of adult *Tribolium*, a species with a CNC, showing the full extent of the free MpT region for one tubule. Note that the perirectal MpTs are obscured by the PNM, but are illustrated in Fig. 1A. A very similar organisation is seen in larvae. (E) Organisation seen in *Tomaspis* (Cercopidae, Hemiptera) (modified from Wigglesworth, 1974*b*), a species with a filter chamber. Arrows show the direction of flow of gut contents. All images are coloured according to labels in D and E. Note that in A–D the most anterior gut structures are omitted. See Fig. 1C for a comparison with lepidopteran larva.

A CNC also appears to have arisen in Diptera, within fungus gnats. One of these is the New Zealand glow worm (Table S1; Green, 1980; Wheeler & Williams, 1915). The larva has a sophisticated CNC, with similarities to that seen in Coleoptera. The rectal epithelium is closely surrounded by visceral muscle, and the MpTs are bound to this by a perinephric membrane. Although the MpTs all lie on one side of the complex, the perinephric membrane ensheaths the entire circumference of the CNC. Curiously the rectal epithelium is thicker on the side facing the MpTs and shows features indicating a transport function, apparently contributing to the likely absorptive role of this structure (Green, 1980). This agrees with the apparent situation in Lepidoptera, where measurements of ion concentrations suggested active transport across the rectal epithelium (Ramsay, 1976), but in contrast to in Coleoptera where absorption may be driven solely by MpTs within the CNC. Within the MpTs, very thin cells face the perinephric membrane. These have been speculated to be leptophragmata‐like cells, although there is no thinning of the perinephric membrane overlying them. Although there is no knowledge of the permeability of the perinephric membrane to Cl^−^ in this species, it is possible that these are rudimentary leptophragmata with functions akin to those in Coleoptera (Green, 1980). Curiously the light organs of *Arachnocampa* species appear to be a specialised posterior portion of the cryptonephridium (Rigby & Merritt, 2011; Green, 1980; Wheeler & Williams, 1915), possibly because the CNC is already a site of high metabolic activity. A rectal complex is also found in the larva of another fungus gnat, *Keroplatus testaceus* (Stammer, 1932; Fig. 4A).

#### (d) Occurrence in Hymenoptera

CNCs appear to have arisen twice within Hymenoptera (Table S1). In many sawflies (suborder Symphyta) MpTs associated with the rectum, and a perinephric membrane, have been observed. A wide spectrum of conditions have been identified including some species with only a loose binding of the distal MpTs to the rectum, and some in which the tubules do not associate with the rectum at all (Maxwell, 1955).

In the fire ant *Solenopsis saevissima* (family Formicidae), the distal ends of the MpTs are very closely associated with the rectal epithelium. An extracellular matrix forms basal laminae, which surrounds both the MpTs and rectum, and these laminae appear to fuse together at the interface between the MpT and rectal epithelia. In places, muscles sit between the two epithelia, separating the laminae. *S. saevissima* has no perinephric membrane (Arab & Caetano, 2002), so this can be considered a simpler rectal complex. *S. saevissima* can survive in conditions and upon diets providing limited water, so their rectal complex may be an adaptation for water conservation (Arab & Caetano, 2002).

#### (e) Occurrence in Neuroptera

A CNC also appears to have arisen once within the order Neuroptera (net‐winged insects). Neuroptera with terrestrial larvae have a CNC (Aspöck & Aspöck, 2007), whilst aquatic larvae of the family Nevrorthidae have no CNC (Beutel, Friedrich & Aspöck, 2010; Aspöck & Aspöck, 2007), suggesting a water‐conservation role in the terrestrial species. In the aquatic larvae of family Sisyridae, only a single MpT is associated with the rectum, which has been argued to indicate that it had a terrestrial ancestor (Beutel *et al*., 2010; Aspöck, Plant & Nemeschkal, 2001). Larval *Acanthaclisis* (ant lion, family Myrmeleontidae) is one of the terrestrial forms for which the CNCs structure has been studied. It has a perinephric membrane which surrounds the distal MpTs associated with the rectum. Two sections of its CNC have been described, a thinner section, and a thicker section in which a portion of the normal perinephric membrane is replaced by a double epithelial layer (Poll, 1936; Loziński, 1911), however the relevance of this curious form remains unexplored.

#### (f) Occurrence in hemimetabolous insects

The cases detailed above relate to CNCs that appear to have evolved independently approximately seven times, some of which have progressed into highly sophisticated forms. All are in holometabolous insects, that is those with a complete metamorphosis from their larval stage. Outside this group a few scattered examples of CNCs or more rudimentary rectal complexes are known to exist.

Western flower thrips (*Frankliniella occidentalis*), from the order Thysanoptera, have a rectal complex (Table S1), although it appears highly rudimentary. Two of their four MpTs run alongside the hindgut, with their distal sections adhering to the rectal pads. They have no perinephric membrane. The epithelia of the MpTs and rectal pads are only separated by a basal lamella. It has been argued that this rectal complex is likely to fulfil a fluid‐absorption role, as the MpT epithelium in proximity to the rectal pads is rich in mitochondria and membrane infoldings (Dallai, Del Bene & Marchini, 1991; Ullman *et al*., 1989).

Species of leafhopper, cicada and frog hopper, from the order Hemiptera, are reported to have the distal ends of their MpTs attached to their rectum and ensheathed in a fine perinephric membrane (Table S1). These species also possess filter chambers (see Fig. 4E) which are specialised organs consisting of regions of the midgut associated with regions of MpT, and are a means of rapidly removing excess water from the sap they ingest, and expelling it into the hindgut. Their rectal complexes cannot be for conservation of water as these species are rather faced with the problem of excessive water in their diet, and are more likely to relate to the conservation of salts (Goodchild, 1966, 1988; Dai *et al*., 2019).

#### (g) Occurrence in myriapods

The millipede *Polyxenus lagurus* appears to be the only arthropod outside the insects known to possess a CNC (Table S1). It is unusual in that central rather than distal sections of the MpTs are associated with the rectum (Schlüter & Seifert, 1985). It has a perinephric membrane, and this is formed from several layers of flattened cells, possibly originating from the mesoderm. Within its CNC the walls of the MpTs are substantially thickened, and cells display many membrane infoldings and elongated mitochondria, suggestive of high levels of transport through this epithelium (Schlüter & Seifert, 1985). *P. lagurus* can produce faeces with relatively low water content, and it has been argued that as it feeds on lichens which become desiccated in times of drought, the CNC could be an adaptation to survive fluctuations in water availability. Similar to tenebrionid beetles, *P. lagurus* can absorb water when the relative humidity of the surrounding atmosphere exceeds 85% (Wright & Westh, 2006).

### (2) Diet, environment and CNCs

Support for a widespread role of CNCs in water conservation is provided by a correlation between aquatic lifestyle and CNC loss. It has been lost in aquatic or wetland larvae, such as in the beetles *Donacia* and *Plateumaris* (family Chrysomelidae) and *Notaris* (superfamily Curculionoidea) (Ivie, 1985; Stammer, 1934; Table S1; Poll, 1932). The semiaquatic beetle *Galerucella* (family Chrysomelidae) has been interpreted as having a poorly developed CNC with a very thin perinephric membrane (Saini, 1964). Absence of a CNC was also noted in larvae of the beetle subfamily Platypodinae (ambrosia beetles) within superfamily Curculionoidea (Stammer, 1934; Conet, 1934), which are mostly species that feed on ambrosia fungus within tree trunks. These species tend to be found in the wet tropics, so it is possible there is no selective pressure for highly efficient water conservation in this group. In the lepidopteran clade Apoditrysia, which generally have a CNC, aquatic larvae of some moths from the family Crambidae lack a CNC (for example *Parapoynx*, *Cataclysta* and *Nymphula*) (Wigglesworth, 1974*b*; Saini, 1964). By contrast, in the coleopteran superfamily Byrrhoidea‐2 (Fig. 3A), CNCs may only occur in adults of some aquatic species, and not in their terrestrial larvae, and are absent in more‐terrestrial species (Hinton, 1939, 1941; Hinton & Matthews, 1955). However, the possession of CNCs in this clade remains unclear (see Section (a)).

For the New Zealand glow worm (Diptera), which is found where water is plentiful, conservation of water may not be the primary purpose of the CNC, and it may rather reflect adaptation to its diet (Green, 1980). However, it has been suggested that the CNC in this species may be important for water conservation during periods of fasting, in order to produce sufficient amounts of mucus to capture its prey (Rigby & Merritt, 2011).

*Onymacris* are tenebrionid beetles of the Namib desert, which have been studied with respect to harvesting of water from coastal fog. *Onymacris unguicularis* performs fog basking, in which they collect water from fog on their body surface using specialised cuticular adaptations. This water trickles into the mouth (Hamilton & Seely, 1976). Desert tenebrionid beetles feed on detritus with only 1–8% water content which is too low to maintain their foraging behaviour and egg laying. For *O. unguicularis* and other species adapted to drinking condensed fog, this is their main source of water intake, and is necessary to maintain water balance over longer time periods (Seely, 1979; Cooper, 1982). The CNC is thus likely an important water‐conservation adaptation for these desert beetles. The Namib desert has a surprisingly high relative humidity for parts of the day, even in the absence of fog (Mitchell *et al*., 2020), and it is conceivable that they can absorb this water vapour using their CNC. Second‐ and third‐instar larvae of *Onymacris plana* and *O. marginipennis* can gain water when placed in high‐humidity conditions, and very little of this could be accounted for by metabolically produced water (Coutchié & Crowe, 1979).

*O. marginipennis* has a lower water absorption threshold and higher absorption rate than *Tenebrio* (Coutchié & Machin, 1984), correlating with a higher surface area of their CNC (Machin & O'Donnell, 1991; Chapman, 1998*b*; see Section (a)). Its highly efficient CNC thus could be an important adaptation allowing these beetles to survive in their desert habitat, although vapour absorption in their natural environment and its significance for their survival has yet to be clearly established (Mitchell *et al*., 2020). The CNCs of *Onymacris* species are more heavily tracheated than those of *Tenebrio*, consistent with a greater ability of *Onymacris* to conserve water compared to *Tenebrio* as an adaptation to survival in the desert (Machin & O'Donnell, 1991; see Section (c)).

CNCs are also common pests of stored food or other organic materials with a very low water content. These include *Tribolium*, which is a major pest of stored grain products, and many other beetles with CNCs including multiple woodworm species, the lesser grain borer *Rhyzopertha dominica*, wheat and rice weevils, and the varied carpet beetle *Anthrenus verbasci*. Invasion of human food stores by many of these beetles dates back at least to Ancient Egypt (Panagiotakopoulos, Buckland & Kemp, 2010). Within Lepidoptera, such pests include the cacao moth *Ephestia elutella*, Mediterranean flour moth *Ephestia kuehniella*, Indian meal moth *Plodia interpunctella* and common clothes moth *Tineola bisselliella*.

Whilst current evidence supports a primary role of coleopteran CNCs as adaptions for water conservation, in lepidopterans the CNCs appear to have other important functions, for example acid–base balance and recycling of other ions. It may be that the CNCs of these different groups are only superficially similar at a structural level and serve quite distinct functions. Alternatively CNCs may serve several functions, with one or another of these more predominant in each group.

The widespread loss of CNCs in aquatic species provides the strongest evidence that CNCs may primarily function in conservation of water, even in groups such as Lepidoptera where additional functions in recycling of ions seem likely. An alternative explanation is that CNC loss in aquatic insects results from adaptations to remove excess water in these species.

Phytophagy has been described as a major evolutionary hurdle, particularly adoption of external feeding on leaves (Mitter, Farrell & Wiegmann, 1988; Southwood, 1972; Strong, Lawton & Southwood, 1984). It is noteworthy that external folivory in insects has arisen within groups possessing CNCs (Table S1; Fig. 3). This includes within Lepidoptera (Barbehenn & Kristensen, 2003; Powell, Mitter & Farrell, 1998; Mitter & Winkler, 2008), Phytophaga within the Cucujiformia infraorder of beetles (Crowson, 1981; Dufour, 1824; Farrell, 1998; Saini, 1964; Mitter & Winkler, 2008), and Symphyta (the sawflies) within Hymenoptera (Mitter & Winkler, 2008; Maxwell, 1955). Such external folivores are exposed to the environment, and may therefore be more vulnerable to desiccation (Connor & Taverner, 1997; Southwood, 1972; Strong *et al*., 1984). Species with a CNC are expected to have an enhanced ability to conserve water, potentially allowing them to overcome this evolutionary hurdle.

In larval Lepidoptera, there is evidence for a major role of the CNC in ion recycling. Many species feed on leaves, and generate extremely alkaline midgut contents to allow efficient digestion. Their CNCs are thought to be critical for the recovery of ions such as HCO_3_^−^ which generate this alkalinity, and cycling of ions could also function in the removal of plant toxins (Kolosov & O'Donnell, 2019*a*). However, Lepidoptera larvae of the basal groups Heterobathmioidea, Neopseustoidea and Eriocranioidea, which likely lack a CNC (Fig. 3B), are generally leaf borers. CNCs may therefore have allowed larvae to overcome the evolutionary hurdle posed by external folivory, rather than folivory *per se*, although a role in reclaiming ions may have enabled more efficient leaf digestion and faster growth.

### (3) Importance of CNCs during larval stages

In Section (2) we argued that CNC loss is generally correlated with an aquatic lifestyle, or a diet and habitat with ample water. In these cases it is generally only the larval stages that are aquatic, with CNCs apparently lost despite the terrestrial life of adults. In addition, in some species the larval CNC is lost in the adult. These include all Lepidoptera which have a CNC (Kolosov & O'Donnell, 2019*a*; Judy, 1968), and appears also to be the case for Neuroptera (Quartey & Kumar, 1973; van Zyl & van der Linde, 2000; Poll, 1936; Loziński, 1911; Table S1). These observations suggest that the CNC may primarily be an adaptation for larval life. One explanation for the apparent prevalence, and often higher sophistication, of CNCs in holometabolous insects compared to hemimetabolous insects and other arthropod groups, is that they are an adaptation to the period of rapid feeding and growth of the larval stages of many holometabolous insects. Indeed Ramsay (1964, 1976) noted that CNCs are usually found in species with a rapid throughput of food. This could make maintenance of water and salt balance a significant challenge when they are secreted into the midgut for digestion, which appears to be the case in *Tenebrio* and lepidopteran species (Ramsay, 1964, 1976).

### (4) Forms of CNCs

#### (a) Extent of CNC upon the rectum

The extent of the hindgut occupied by the CNC can differ significantly among taxa. For example, the boursouflures in *Onymacris* are much smaller and more closely packed than in *Tenebrio*, creating a much larger surface area for transport (Machin & O'Donnell, 1991). This correlates with a greater ability to absorb water in *Onymacris* than *Tenebrio* (see Section (2)).

In laterally displaced CNCs, the MpTs and enveloping perinephric membrane are situated only on one side of the hindgut (Fig. 4B). Examples of this form include beetle species of the superfamily Bostrichoidea (Table S1), the family Mycetophagidae (hairy fungus beetles) within the superfamily Tenebrionoidea, and *Sitophilus* species within the superfamily Curculionoidea (Table S1). Based upon the diet and habitat of species with laterally displaced CNCs, it has been proposed that they could be more efficient at recovering water (Saini, 1964), although this seems counterintuitive. The Bostrichoidea species with laterally displaced CNCs are adapted to dry conditions and diets with little water, including stored grain and wood (Saini, 1964), and *Sitophilus* are weevils feeding on dry stored food. However, of the Mycetophagidae species, *Typhaea* feeds on mould associated with ripening hay and unharvested grain crops, and *Mycetophagus multipunctatus* feed on tree fungi, which may be expected to provide higher water content.

In the case of dipteran CNCs, whilst the MpTs are only situated on one side of the rectum in *Arachnocampa* species (glow worms), they completely surround the rectum in the fungus gnat *Keroplatus testaceus* (Fig. 4A; Wheeler & Williams, 1915; Stammer, 1932) *Arachnocampa* also have a perinephric membrane which *Keroplatus* lacks. *Keroplatus* spp. feed on fungal spores, which presumably have a low water content, whilst New Zealand glow worm larvae are predatory and can access more water from their diet (Stammer, 1932; Rigby & Merritt, 2011; Osawa, Sasaki & Meyer‐Rochow, 2014). This argues against lateral displacement being more efficient for water conservation and that lateral displacement may serve a different purpose. The very distal parts of the cryptonephric MpTs of *Arachnocampa* are modified to form light organs (Rigby & Merritt, 2011; Green, 1980; Wheeler & Williams, 1915). These are closely apposed to a tracheal reflector structure that directs light in a dorsal direction (Green, 1980), and it is possible that the MpTs have become laterally displaced to establish this organisation. Overall it remains unclear whether laterally displaced forms of CNC are more or less efficient, and what has driven their evolution in different insect groups.

#### (b) Presence of leptophragmata

The leptophragmata may have evolved once within Coleoptera, as a specialised route for K^+^ and Cl^−^ transport from the haemolymph into the CNC. Recent findings from *Tribolium* show leptophragmata to be critical for the physiological functions of the CNC in this species (Section (b)). Leptophragmata are found in the superfamily Tenebrionoidea (Table S1). *Onymacris* species of this superfamily have leptophragmata, although with no overlying blister (Machin & O'Donnell, 1991). Leptophragmata have also been observed in species within the superfamilies Chrysomeloidea, Cucujoidea, Curculionoidea, and Bostrichoidea (Table S1). In *Onymacris*, as well as having smaller, more tightly packed boursouflures compared to *Tenebrio*, it has boursouflures throughout its CNC, whilst in *Tenebrio* they only occur in the posterior two thirds of the CNC, with the result that *Onymacris* (which has a greater ability to absorb water than *Tenebrio*) has about 6.3 times more boursouflures (Machin & O'Donnell, 1991). As each boursouflure is expected to bear a single leptophragma cell, a correlation between leptophragmata abundance and water absorption ability would support the centrality of leptophragmata in this process.

In some species with laterally displaced CNCs, including adult *Anthrenus vorax*, it was suggested that the leptophragmata are restricted to certain portions of the cryptonephridial MpTs (Saini, 1964). We could identify only a handful of leptophragmata (using silver nitrate staining) in adult *Anthrenus verbasci* (varied carpet beetle), at the posterior end of the CNC (Fig. S1), associated with a more anterior loop of the hindgut (Figs S1 and 4B). The functional significance of this reduced and spatially restricted population of leptophragmata remains unexplored, as does the lateral displacement of CNCs more generally.

In the family Ptinidae (which include wood‐boring beetles, and pests of stored food and medicine), within Bostrichoidea, leptophragmata are reportedly absent, despite these species living on dry food. Here the cryptonephridial tubules do not closely associate with the perinephric membrane, leading Saini (1964) to suggest that leptophragmata may play a structural role, attaching the MpTs to the perinephric membrane. Species from Ptinidae have laterally displaced CNCs with similar organisation to that of *Anthrenus* (Saini, 1964), and it is possible that they have a small population of leptophragmata that were not captured in histological observations.

The common cockchafer *M. melolontha* from the superfamily Scarabaeoidea has a rectal complex, but leptophragmata appear to be absent (Lison, 1938; Saini, 1964), supporting the independent evolution of the rectal complex in this group.

In Lepidoptera the cryptonephridial MpTs do not form contacts with the perinephric membrane, and leptophragmata are absent (Ramsay, 1976). There is evidence for ion transport from both the rectal contents, and the haemolymph, into the cryptonephridial tubules in Lepidoptera (Kolosov & O'Donnell, 2019*a*). It is likely that distinct transport mechanisms exist in this group to transport ions across the perinephric membrane in the absence of leptophragmata.

### (5) Countercurrent organisation of the CNC maximises its efficiency

The vertebrate nephron is highly efficient at generating concentrated excreta, with its countercurrent system underpinning extremely efficient exchange mechanisms (Pallone *et al*., 2003; Sands & Layton, 2009). At its most efficient, this represents a crucial adaptation to minimise water loss in desert vertebrates (Dantzler, 1982). It is notable that the osmoregulatory systems of most arthropods, including the MpTs and hindgut, typically do not have such an organisation. This is resolved in the rectal pads of *Calliphora* (blowfly), by generating higher ionic concentrations in intercellular spaces than in the cytoplasm of the rectal epithelium to establish a very local osmotic gradient which is thought to drive water transport from the rectal lumen to the haemolymph (Gupta *et al*., 1980). The CNC represents an important exception, as the close proximity of the MpTs and rectum, with an opposing direction of flow of their lumenal contents, creates a countercurrent system (Phillips, 1970; Kirschner, 1967).

### (6) Malpighian tubules and gut form integrated systems, predisposed to evolve into a CNC

Epithelia generate local fluxes in water and ions, so their position relative to other organs is critical to their function. Species with CNCs display great variation in the extent of the coupling of the MpTs to the rectum. Furthermore, spatial relationships between regions of the MpT and gut also exist in species lacking a CNC, even where no direct physical connection exists. Ancestral functions of the rectum and distal tubule, as well as existing spatial relationships, may therefore predispose them to establishing CNCs, which could explain how this organisation has evolved so many times independently.

MpTs are long and convoluted structures, but their arrangement is generally highly stereotypical within a species (Wigglesworth, 1974*b*). There is evidence that their position is functionally important, as mispositioning of MpTs in *Drosophila* by genetic manipulation led to fluid retention in the body cavity and premature death (Weavers & Skaer, 2013). In *Drosophila* there are differences in the developmental identity and expression profiles of the anterior pair of MpTs, the distal ends of which lie close to the anterior midgut, and the posterior pair of MpTs, whose distal ends lie alongside the rectum (Fig. 4C; Hatton‐Ellis *et al*., 2007; Chintapalli *et al*., 2012). These observations support the notion that MpT positioning within the body is of functional importance, including in species without a CNC arrangement.

In species without a CNC, the rectum is able to absorb water and solutes, including Cl^−^ from the gut lumen into the haemolymph (Wigglesworth, 1932, 1974*b*; Ramsay, 1952; Bone & Koch, 1942; Gupta *et al*., 1980; Buxton, 1932). It has also been shown in insects without CNCs, including *R. prolixus* and *Drosophila*, that secretion into the MpT lumen often occurs in the distal regions of the MpTs, and reabsorption occurs in the proximal regions (Wigglesworth, 1931*b*; O'Donnell & Maddrell, 1995). Thus, there could be a natural benefit of coupling the absorptive rectum with the secretory distal MpTs.

Common patterns of MpT architecture can be observed across disparate insects. The MpTs, which enter the gut at the midgut–hindgut boundary, generally extend anteriorly into the animal, often in close proximity to the midgut. They then often fold back upon themselves and run posteriorly, and their distal ends lie close to the rectum. This general pattern is seen in many species with rectal complexes including coleopteran species such as *Alphitobius diaperinus* (McAllister, Steelman & Carlton, 1995), *Tribolium* (King & Denholm, 2014; Fig. 4D), *Anthrenus* (Saini, 1964; Fig. 4B; Mobüsz, 1897), *Galerucella birmanica* (Khatib, 1946), and *M. melolontha* (Lison, 1938), lepidopteran species such as *Trichoplusia ni* (cabbage looper) (O'Donnell & Ruiz‐Sanchez, 2015; Fig. 1C), and the dipteran fungal gnat *K. testaceus* (Stammer, 1932; Fig. 4A).

A similar pattern is also seen in species without a rectal complex including some dipteran mosquito larvae such as *Anopheles gambiae* (Linser *et al*., 2009) and *Aedes aegypti* (Wigglesworth, 1974*b*), several species from the order Hemiptera (Goodchild, 1963, 1966), including *R. prolixus* (Wigglesworth, 1931*a*), the order Siphonaptera, for example the rat flea *Xenopsylla cheopis* (Bernotat‐Danielowski & Knülle, 1986), and the order Phasmatodea (stick insects), for example *Aretaon asperrimus* (Shelomi & Kimsey, 2014; Shelomi, 2017), *Leptynia attenuata* (Sinéty, 1901) and *Carausius morosus* (Ramsay, 1953*a*). A similar organisation is also seen for the posterior tubule pair in *Drosophila*, but not the anterior pair (Fig. 4C). Lastly a similar pattern is seen in some groups of Hemiptera (true bugs), such as the suborders Auchenorrhyncha and Sternorrhyncha which have a filter chamber. In these species a complex arrangement exists, where the posterior midgut along with proximal sections of the MpTs are enclosed in an invagination in the anterior midgut (Fig. 4E). This structure enables some of the excessive water present in the xylem sap which these insects eat, to bypass the midgut, so a more concentrated fluid can pass into the midgut for digestion. Again the distal MpT regions tend to lie in proximity to the rectum (Edney, 1957; Goodchild, 1966, 1988; Wigglesworth, 1974*b*).

Given that the MpTs insert at the boundary between the midgut and hindgut, that the gut contents flow from an animal's anterior to its posterior, and that the MpT contents flow from their blind distal ends to their proximal openings into the gut, this configuration is a simple means to achieve a recycling system for the gut, with water and solutes passing through epithelial layers allowing selectivity and regulation of exchange. In lepidopteran larvae, the proximal section of the MpTs is closely applied to the surface of the midgut and there is evidence that water reclaimed from the rectal lumen by the CNC flows along the MpTs and is then transferred to the midgut lumen (Kolosov & O'Donnell, 2019*a*; Reynolds & Bellward, 1989; Fig. 1C). In Hemipteran species with filter chambers, the movement of water out of the lumen of the gut at the posterior foregut/anterior midgut is driven by an osmotic gradient. To establish this, the MpTs secrete potassium chloride into their lumen in their free distal section, and this flows into the proximal part of the MpTs within the filter chamber. This fluid containing high levels of potassium chloride drains into the anterior end of the hindgut, and K^+^ and Cl^−^ are reabsorbed from the hindgut into the haemolymph, allowing these ions to cycle continuously within the animal (Hubert *et al*., 1989; Cheung & Marshall, 1973; Marshall, 1983; Chapman, 1998*a*).

The most striking exception to this pattern is seen in many dipteran species, where an anterior pair of MpTs have their distal ends close to the midgut. This includes in *Ptychoptera* (Wigglesworth, 1974*b*), *Lucilia cuprina*, *Drosophila hydei* and *D. melanogaster* (Fig. 4C) and *Musca autumnalis*. In these species the distal parts of the anterior MpTs contain many granules of calcium salts (Waterhouse, 1950; Wessing, Zierold & Hevert, 1992; Grodowitz, Broce & Kramer, 1987; Wigglesworth, 1974*b*). These MpT regions are thought to play an important role in sequestering calcium (Chintapalli *et al*., 2012), which is generally absorbed by the insect midgut (Wood & Harvey, 1976; Taylor, 1987). Calcium absorption from the midgut contents may be required to generate the optimal conditions for digestion, whilst calcium sequestration prevents it reaching toxic levels in the haemolymph and may also act as a reserve in case of calcium scarcity (Taylor, 1987). A calcium‐handling function could therefore explain this distinct MpT architecture.

The typical organisation, in which the proximal MpT regions loop anteriorly in proximity to the midgut, with the distal regions extending to surround the rectum, could evolve readily into a rectal complex by establishment of a more intimate association between the distal tubules and rectum. It would also align the MpTs and rectum in an orientation where a countercurrent system could readily be established. Evolution of a perinephric membrane may not have fundamentally altered the function of the rectal complex, but by insulating the CNC from the haemolymph, likely produced a step change in the efficiency with which it could operate. As seen in *Tenebrio*, the fluid in the very distal ends of the MpTs within its CNC can reach extremely high ionic concentrations and this could only be achieved by insulating this compartment from the haemolymph. These transitions would be akin to other evolutionary transitions such as the multiple times closed circulatory systems have arisen from open systems (Monahan‐Earley, Dvorak & Aird, 2013), or the multiple occurrences that myelin sheaths have been gained in nervous systems, from the loose glial covering observed in the ancestral state (Knowles, 2017; Stiefel, Torben‐Nielsen & Coggan, 2013; Hartline, 2011; Monahan‐Earley *et al*., 2013).

## V. FUTURE DIRECTIONS

Recent advances have opened up possibilities to investigate CNC biology using molecular approaches. This is fertile ground for answering important developmental, physiological and evolutionary questions, and to understand their relevance in a wider ecological context. We have discussed how advances in the model beetle species *Tribolium* are enabling these questions to be answered (Naseem *et al*., 2023; King & Denholm, 2014; R. Beaven, K.V. Halberg & B. Denholm, in preparation; Beaven *et al*., 2024*b*). These studies in *Tribolium* complement investigations of the lepidopteran CNC (Azuma *et al*., 2012; O'Donnell & Ruiz‐Sanchez, 2015; Audsley *et al*., 1993; Liao *et al*., 2000; Moffett, 1994).

Advances in transcriptomic approaches are also playing an important role in our exploration of this organ system. BeetleAtlas is a *Tribolium* database of gene transcript expression, at different embryonic stages and for specific larval and adult tissues including the CNC/rectal complex (Naseem *et al*., 2023; BeetleAtlas.org; Leader, Naseem & Halberg, 2024). This database is linked with gene information in the genomic resource, iBeetle‐Base, as well as to transcript expression data for orthologous genes of *Drosophila*, in FlyAtlas2. BeetleAtlas has proved of great value, informing exploration of CNC function, such as the role of the NHA1 ion antiporter (Naseem *et al*., 2023). In the future our transcriptomic understanding of the CNC of *Tribolium* will be enhanced by single‐cell sequencing of this organ during development and in its mature form. This will also provide a useful comparison, for example with recent single‐cell sequencing data from the mature renal organs of *Drosophila* (Xu *et al*., 2022).

Finally, an accelerating release of sequenced genomes such as by the Darwin Tree of Life Project (Darwin‐Tree‐of‐Life‐Project‐Consortium, 2022), is opening up the possibility to explore CNCs using molecular approaches in diverse arthropod groups, and to address questions of convergent evolution, as well as commonality and diversity in CNC function.

## VI. CONCLUSIONS

(1) The CNCs of tenebrionid beetles are considered to function primarily in recycling of water from the gut contents back into the body, a mechanism for water conservation that allows survival in extremely dry environments. This is likely to have enabled colonisation of deserts, as well as exploitation of dry food stores by pest species. Our understanding of CNC function is rooted in descriptions of tissue, cell and subcellular structure, and physiological analysis of this system in the mealworm (*Tenebrio*). This is being extended by examining the system from a molecular perspective in the model beetle species, *Tribolium*.
(2) The perinephric membrane tissue, which insulates the CNC to prevent uncontrolled exchange of water and ions with the haemolymph, appears to be an evolutionarily novel tissue found only in CNCs. The CNC as an organ system is also an evolutionary novelty, generated by assembly of preexisting organs and tissues, along with recruitment of the perinephric membrane tissue. Developmental studies in *Tribolium* are revealing the mechanisms underpinning the embryonic formation of the CNC for the first time, and future comparisons of these findings with species lacking a CNC, or with species that have evolved CNCs independently, will shed light on how these novelties evolved.
(3) CNCs have evolved independently multiple times in arthropods. Analysis of their reported phylogenetic occurrence suggests that they have emerged most often within holometabolous insects (i.e. those with complete metamorphosis), although CNCs have also arisen within hemimetabolous insects, and once within myriapods. There is evidence that they are principally important for larval stages, perhaps due to the high throughput of food during larval life, which necessitates recycling of water and solutes from the digestive contents. This could explain the prevalence of CNCs in holometabolous insects. Molecular studies will also shed light on the physiological functions of CNCs from diverse groups, which may be linked to the ecology of these species.
(4) In addition to reclaiming water, there is evidence that CNCs can function to recycle solutes. This has been demonstrated most clearly for larval Lepidoptera, where ions including bases are secreted into the midgut to facilitate digestion, and the CNC functions in their return to the haemolymph. We still lack an understanding of whether CNCs have a unifying primary function, however the loss of CNCs in many aquatic groups, including larval Lepidoptera, could suggest a primary role in water conservation.
(5) Ancestral properties of the MpT and rectal epithelia, in which the distal MpTs are typically secretory and the rectum reabsorptive, could functionally predispose them for the formation of a CNC. So too could architectures of the MpTs in relation to the gut typically seen in species without CNCs, in which the proximal MpT regions are in proximity to the midgut, and distal MpT regions to the rectum. This organisation may be predisposed to the establishment of a countercurrent system.

## Supporting information


**Fig. S1.** Silver staining of leptophragmata in adult *Tribolium castaneum* and *Anthrenus verbasci*.

**Table S1.** Phylogenetic distribution of cryptonephridial complexes (CNCs) and rectal complexes across arthropods.
