# Supplementary material for: The cryptonephridial/rectal complex: an evolutionary adaptation for water and ion conservation
Source: Biol Rev Camb Philos Soc. 2024 Oct 22;100(2):647–71. doi: 10.1111/brv.13156 (PMC11885702; doi:10.1111/brv.13156)
Supplement: Supplementary file 1 — Fig. S1. Silver staining of leptophragmata in adult Tribolium castaneum and Anthrenus verbasci. [file BRV-100-647-s002.docx]

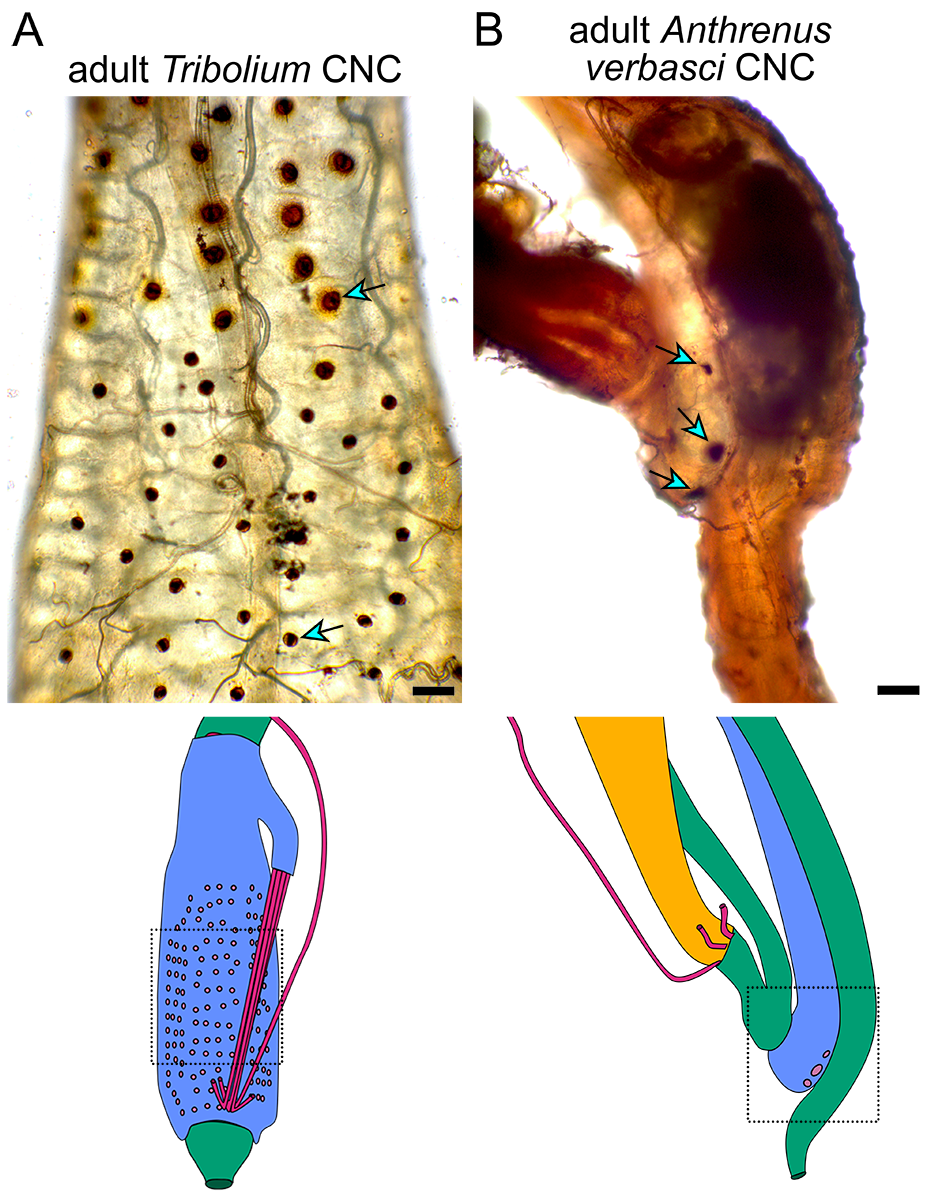


**Fig. S1.** Silver staining of leptophragmata in adult *Tribolium castaneum* and *Anthrenus verbasci*. (A) Dissected cryptonephridial complex (CNC) from a *Tribolium* adult (approximate region imaged shown by dotted square in diagram below), incubated for a few minutes in 1% silver nitrate, which reacts with Cl^–^ in leptophragmata to produce a dark stain. Example leptophragmata are indicated with arrows. (B) Dissected CNC of adult *Anthrenus verbasci* (which is laterally displaced), stained as in A to reveal Cl^–^-rich leptophragmata (arrows). Schematics below are coloured as in Fig. 4B, D. Scale bars = 50µm.
