# Supplementary material for: The cryptonephridial/rectal complex: an evolutionary adaptation for water and ion conservation
Source: Biol Rev Camb Philos Soc. 2024 Oct 22;100(2):647–71. doi: 10.1111/brv.13156 (PMC11885702; doi:10.1111/brv.13156)
Supplement: Supplementary file 2 — Table S1. Phylogenetic distribution of cryptonephridial complexes (CNCs) and rectal complexes across arthropods. [file BRV-100-647-s001.docx]

**Table S1.** Phylogenetic distribution of cryptonephridial complexes (CNCs) and rectal complexes across arthropods. Some beetle groups are numbered, e.g. Byrrhoidea-2, according to the nomenclature of McKenna *et al.* (2019). MpT, Malpighian tubule.

| **Class** | **Order** | **Group** | | | | | | | | | **Examples of species studied** | **Comments** |
| --- | --- | --- | --- | --- | --- | --- | --- | --- | --- | --- | --- | --- |
| Insecta | Coleoptera | superfamily Tenebrionoidea | family Tenebrionidae | | | | | | | | *Tenebrio molitor* (mealworm) (Dufour, 1824; Conet, 1934; Saini, 1964; Ramsay, 1964; Grimstone *et al*., 1968), *Blaps mucronata* (churchyard beetle) (Conet, 1934), *Tribolium castaneum* (red flour beetle) (King & Denholm, 2014), *Onymacris plana* and *marginipennis* (fog basking beetles) (Machin & O'Donnell, 1991), *Gnaptor spinimanus* (Gorka, 1914) | CNC with perinephric membrane and leptophragmata present in larvae and adults |
|  |  |  | family Mycetophagidae | | | | | | | | *Typhaea* (Stammer, 1934), *Mycetophagus multipunctatus* (Saini, 1964), *Mycetophagus quadripustulatus* (Hinton, 1941) | laterally displaced CNC present in larvae and adults |
|  |  |  | family Oedemeridae | | | | | | | | *Ischnomera ruficollis* (Dufour, 1824) | has CNC (observed in adult stage) |
|  |  |  | family Mycteridae | | | | | | | | *Mycterus curculioides* (Dufour, 1824) | has CNC (observed in adult stage) |
|  |  |  | family Meloidae | | | | | | | | *Berberomeloe majalis*, *Mylabris quadripunctata*, *Zonitis flava*, *Sitaris muralis* (Dufour, 1824) | has CNC (observed in adult stage) |
|  |  |  | family Mordellidae | | | | | | | | *Mordella fasciata* (Dufour, 1840) | CNC in larvae and adults |
|  |  | superfamily Bostrichoidea | family Bostrichidae | | | | | | | | *Rhyzopertha dominica* (lesser grain borer) (Saini, 1964) | laterally displaced CNC with perinephric membrane, and leptophragmata (observed in adult stage) |
|  |  |  | family Dermestidae | | | | | | | | *Dermestes lardarius* (larder beetle) (Saini, 1964), *Attagenus pellio*, *Anthrenus museorum* (museum beetle) (Conet, 1934), *Anthrenus verbasci* (varied carpet beetle) (Mobüsz, 1897), and *Anthrenus flavipes* (furniture carpet beetle) (Saini, 1964) | laterally displaced CNC in larvae and adults, with perinephric membrane, and leptophragmata |
|  |  |  | family Ptinidae | | | | | | | | *Ptinus tectus* (Australian spider beetle), *Niptus hololeucus*,  *Stegobium paniceum*, *Lasioderma serricorne* (Saini, 1964) | laterally displaced CNC with perinephric membrane. No leptophragmata (observed in adult stage) |
|  |  | superfamily Chrysomeloidea | family Chrysomelidae | | | | | subfamily Galerucinae | | | *Xanthogaleruca luteola* (elm leaf beetle) (Poyarkoff, 1910), *Agelastica alni* (alder leaf beetle) (Conet, 1934), *Altica bimarginata* (Woods, 1916), *Galerucella birmanica* (Khatib, 1946; Saini, 1964) and *G.* *tenella* (strawberry leaf beetle) (Saini, 1964), *Lochmaea crataegi* (hawthorn leaf beetle) (Saini, 1964) | CNC with fine perinephric membrane (originally interpreted as muscle layer) and leptophragmata in larvae and adults. Saini (1964) comments that the semi-aquatic *Galerucella* has a poorly developed cryptonephridial system |
|  |  |  |  |  |  |  |  | subfamily Chrysomelinae | | | *Gastrophysa viridula* (green dock beetle), *Chrysomela cuprea* (Conet, 1934) | CNC with perinephric membrane |
|  |  |  |  |  |  |  |  | subfamily Bruchinae | | | *Bruchus pisi* (pea weevil) (Conet, 1934), *Acanthoscelides obsoletus* (Saini, 1964) | CNC with perinephric membrane and leptophragmata in larvae and adults |
|  |  |  |  |  |  |  |  | subfamily Donaciinae | | | *Donacia* sp. and *Plateumaris* sp. (Stammer, 1934; Poll, 1932) | CNC lost in larvae of these aquatic and wetland groups |
|  |  |  | family Cerambycidae | | | | | | | | *Anaglyptus mysticus* (Saini, 1964) | CNC with perinephric membrane and leptophragmata |
|  |  | superfamily Cleroidea | | | | | | | | | *Malachius aeneus*, *Trichodes alvearius*, *Thymalus limbatus* (Dufour, 1824) | has CNC |
|  |  | superfamily Coccinelloidea | | | | | | | | | *Epilachna indica* and *Coccinella septempunctata* (five-spot ladybird) (Pradhan, 1942), *Adalia bipunctata* (two-spot ladybird) (Conet, 1934; Saini, 1964) | CNC with thin perinephric membrane and leptophragmata |
|  |  | superfamily Cucujoidea | | | | | | | | | *Cryptolestes pusilloides*, *C*. *pusillus* and *C*. *turcicus* (Saini, 1964) | CNC with perinephric membrane and leptophragmata (observed in adult stage) |
|  |  | superfamily Curculionoidea | family Curculionidae | | | | subfamily Dryophthorinae | | | | *Sitophilus granaries* (wheat weevil) and *Sitophilus oryzae* (rice weevil) (Ivie, 1985; Saini, 1964) | laterally displaced CNC with perinephric membrane and leptophragmata (observed in adult stage) |
|  |  |  |  |  |  |  | subfamily Platypodinae | | | | *Platypus* sp. (ambrosia beetles) (Stammer, 1934) | CNC not seen in larvae. Ambrosia fungus feeders from tropics |
|  |  |  | family Brachyceridae | | | | | | | | *Notaris* sp. (marsh weevils) (Stammer, 1934) | CNC lost in larvae of these aquatic and wetland beetles |
|  |  |  | family Anthribidae | | | | | | | | *Araecerus fasciculatus* (Saini, 1964) | CNC with perinephric membrane and leptophragmata (observed in adult stage) |
|  |  | suborder Adephaga | | | | | | | | | *Carabus auratus* (golden ground beetle), *Aptinus displosor*, *Scarites buparius*, *Clivina arenaria*, *Chlaenius vestitus*, *Laemostenus terricola*, *Pterostichus madidus*, *Zabrus tenebrioides*, *Harpalus rufipes*, *Nebria complanate*, *Omophron limbatum*, *Cicindela campestris*, *Cybister lateralimarginalis*, *Gyrinus natator* (Dufour, 1824) | no CNC observed in adult |
|  |  | superfamily Byrrhoidea-2 | | family Dryopidae | | | | | | | Species including *Dryops* spp. and *Helichus* spp. (Hinton, 1939). *Dryops* spp. (Saini, 1964; Dufour, 1834) | no CNC in larvae. In adult Dufour (1834) reported that there is no CNC, Hinton (1939) that there is a CNC, and Saini (1964) that there is no CNC but that there are two short regions where the MpTs are bound to the gut by a thin membrane |
|  |  |  |  | family Lutrochidae | | | | | | | *Lutrochus montanus* and *L. geniculatus* (Hinton, 1939) | no CNC in larvae. CNC reported in adult stage but findings from family Dryopidae cast doubt on this |
|  |  |  |  | family Psephenidae | | | | | | | *Psephenus palpalis* (Hinton, 1939) | no CNC in larvae or adults |
|  |  |  |  | family Elmidae | | | | | | | *Elmis* sp., *Neoelmis* sp., *Phanoceroides aquaticus*(Hinton, 1939) | no CNC in larvae or adults |
|  |  |  |  | family Limnichidae | | | | | | | *Limnius* sp., *Limnichites* spp., *Ersachus erichsonianus* (Hinton, 1939) | no CNC in larvae or adults |
|  |  | superfamily Buprestoidea | | | | | | | | | *Agrilus viridis* (Dufour, 1824) | no CNC observed in adult |
|  |  | superfamily Byrrhoidea-1 | | | | | | | | | *Byrrhus* *pyrenaeus*, *B. pilule*, *Amphycyrta* sp. (Hinton, 1939) | no CNC in larvae or adults |
|  |  | superfamily Elateroidea | | | | | | | | | *Agrypnus murinus*, *Lygistopterus rubripennis*, *Lamprohiza splendidula*, *Metacantharis clypeata* (Dufour, 1824) | no CNC observed in adult |
|  |  | superfamily Staphylinoidea | | | | | | | | | *Staphylinus erythropterus*, *Paederus riparius*, *Silpha obscura* (Dufour, 1824), *Nicrophorus vespilloides* (Beaven *et al.*, 2024*a*) | no CNC observed in adult (Dufour, 1824), or larval stages (Beaven *et al.*, 2024*a*) |
|  |  | superfamily Hydrophiloidea | | | | | | | | | *Hister* (Dufour, 1824) | no CNC observed in adult |
|  |  | superfamily Scarabaeoidea | tribe Melolonthini | | | | | | | | *Melolontha melolontha* (common cockchafer) (Lison, 1938; Saini, 1964; Beaven *et al.*, 2024*a*) | adults have rectal complex with very fine enveloping cellular layer (possibly a perinephric membrane). No leptophragmata observed. In early report no CNC was observed in adult male (Dufour, 1824). Larvae have small amount of elaborated MpTs associated with rectum (Beaven *et al.*, 2024*a*) |
|  |  |  |  |  |  |  |  |  |  |  | *Amphimallon majale* (European chafer) (Menees, 1958) | rectal complex with enveloping membrane reported in larvae |
|  |  |  |  |  |  |  |  |  |  |  | *Polyphylla decemitheata* (ten- lined June beetle) (Areekul, 1957) | suggested that larvae have distal MpTs bound to surface of rectum by an enveloping membrane |
|  |  |  |  |  |  |  |  |  |  |  | *Phyllophaga anxia* (Berberet & Helms, 1972) | some association of distal MpTs with rectum seen in larvae and adults |
|  |  |  |  |  |  |  |  |  |  |  | *Phyllophaga gracilis* (Areekul, 1957; Fletcher, 1930) | CNC not noted in adults by Fletcher (1930). Areekul (1957) suggested that larvae have distal MpTs bound to surface of rectum by an enveloping membrane |
|  |  |  | tribe Dichelonychini, | | | | | | | | *Dichelonyx lateralis*  (Areekul, 1957) | suggested that larvae have distal MpTs bound to surface of rectum by an enveloping membrane |
|  |  |  | tribe Diplotaxini | | | | | | | | *Diplotaxis liberta* (Jones, 1940) | no CNC reported in adult |
|  |  |  | tribe Rhizotrogini | | | | | | | | *Rhizotrogus aestivus* (Schäfer, 1954) | no CNC reported in larvae and adult |
|  |  |  | subfamily Rutelinae | | | | | | | | *Anomala cuprea* (Nagae *et al.*, 2013) | some association of MpTs with rectum in larvae |
|  |  |  |  |  |  |  |  |  |  |  | *Anomala corpulenta* (Wang *et al*., 2024) | no CNC in larvae |
|  |  |  |  |  |  |  |  |  |  |  | *Popillia japonica* (Swingle, 1930) | rectal complex not observed in larvae or adults |
|  |  |  | subfamily Cetoniinae | | | | | | | | *Cetonia aurata* (rose chafer) | no CNC observed in adults (Dufour, 1824, 1842). Rectal complex occurs in larvae but not adults (Beaven *et al.*, 2024*a*) |
|  |  |  |  |  |  |  |  |  |  |  | *Protaetia cuprea* (Werner, 1926) | in larvae, distal MpTs are elaborated on the surface of the rectum |
|  |  |  |  |  |  |  |  |  |  |  | *Pachnoda marginata* (sun beetle) (Beaven *et al.*, 2024*a*) | in larvae, distal MpTs have tightly packed sinuous course, entirely surrounding rectum. Adults lack rectal complex |
|  |  |  | subfamily Dynastinae | | | | | | | | *Heteronychus aratar* (Sheehan *et al*., 1982) | no CNC observed in larvae or adults, although MpTs surround colon and rectum in adult |
|  |  |  |  |  |  |  |  |  |  |  | *Trypoxylus dichotomus* (Wang *et al*., 2024) | no CNC in larvae |
|  |  |  |  |  |  |  |  |  |  |  | *Oryctes nasicornis* (European rhinoceros beetle) (Bayon, 1981; Sirodot, 1858; Gérard, 1942) | in larvae, the distal MpTs are elaborated and applied to the rectum and to a dilated portion of the hindgut |
|  |  |  | subfamily Scarabaeinae | | | | | | | | *Copris lunaris* (horned dung beetle) (Dufour, 1824), *Digitonthophagus catta* (Verma, 1969) | no CNC observed in adults |
|  |  |  | subfamily Aphodiinae | | | | | | | | *Neocalaphodius moestus* (Verma, 1969) | no CNC observed in adults |
|  |  |  | family Geotrupidae | | | | | | | | *Geotrupes stercorarius* (Saini, 1964) | no CNC observed in adults |
|  |  |  | family Pleocomidae | | | | | | | | *Pleocoma crinita* (Areekul, 1957) | reported that larvae have distal MpTs bound to surface of rectum by enveloping membrane |
|  |  |  | family Glaphyridae | | | | | | | | *Lichnanthe rathvoni* (Areekul, 1957) | reported that larvae have distal MpTs bound to surface of rectum by enveloping membrane |
|  |  |  | family Lucanidae | | | | | | | | *Lucanus cervus* (European stag beetle) (Dufour, 1824; Saini, 1964) | no CNC observed in adults |
|  |  |  |  |  |  |  |  |  |  |  | *Dorcus parallelipipedus* | no CNC observed in larvae (Edwards, 1930; Dufour, 1842) or adults (Saini, 1964) |
|  |  |  |  |  |  |  |  |  |  |  | *Platycerus caraboides* (Beaven *et al.*, 2024*a*) | no CNC in larvae |
|  |  |  | family Passalidae | | | | | | | | *Pentalobus barbatus* (Baker, 1968) | no CNC observed in larvae or adults |
|  | Lepidopt-era | clade Ditrysia | superfamily Tineoidea | | | | | | family Tineidae (clothes moths) | | *Tinea columbariella* (Saini, 1964) | larval CNC with perinephric membrane |
|  |  |  |  |  |  |  |  |  | family Psychidae | | *Clania* sp. (Ishimori, 1924) | larval CNC with perinephric membrane |
|  |  |  | superfamily Yponomeutoidea | | | | | | | | *Yponomeuta evonymella* and *Cerostoma sasakii* (Ishimori, 1924) | larval CNC with perinephric membrane |
|  |  |  | clade Apoditrysia | | | superfamily Tortricoidea | | | | | *Olethreutes mori* (Ishimori, 1924) | larval CNC with perinephric membrane |
|  |  |  |  |  |  | superfamily Pyraloidea | | | | family Crambidae | *Parapoynx stratiotata*, *Cataclysta lemnata* (small china-mark) and *Nymphula stratiotata* (ringed china-mark) (Wigglesworth, 1974*b*; Saini, 1964) | aquatic larvae lacking CNC |
|  |  |  |  |  |  |  |  |  |  | family Pyralidae | *Galleria mellonella* (greater wax moth) (Metalnikov, 1908), *Ephestia kuehniella* (Saini, 1964), multiple species (Ishimori, 1924) | larval CNC with perinephric membrane |
|  |  |  |  |  |  | superfamily Pterophoroidea | | | | | *Pselnophorus vilis* (Ishimori, 1924) | larval CNC with perinephric membrane |
|  |  |  |  |  |  | superfamily Lasiocampoidea | | | | | *Euthrix potatoria*, *Malacosoma neustria* (Ishimori, 1924) | larval CNC with perinephric membrane |
|  |  |  |  |  |  | superfamily Bombycoidea | | | | family Saturniidae | *Hyalophora cecropia* (cecropia moth), *Antheraea polyphemus*, *Samia cynthia* (Judy, 1968), *Caligula japonica*, *Antheraea pernyi* (Ishimori, 1924) | larval CNC with perinephric membrane. Cryptonephric MpTs reportedly degenerate by pupal stage |
|  |  |  |  |  |  |  |  |  |  | family Sphingidae | *Manduca sexta* (tobacco hawk moth) (Judy, 1968; Ramsay, 1976), *Theretra oldenlandiae*, *Acherontia styx* (Ishimori, 1924) | larval CNC with perinephric membrane |
|  |  |  |  |  |  |  |  |  |  | family Bombycidae | *Bombyx mori* (domestic silk moth) (Ito, 1921; Ishimori, 1924) | larval CNC with perinephric membrane. Cryptonephric MpTs reportedly detach and degenerate during pre-pupal stage |
|  |  |  |  |  |  |  |  |  |  | family Eupterotidae | *Apha subdives* (Ishimori, 1924) | larval CNC with perinephric membrane |
|  |  |  |  |  |  |  |  |  |  | family Brahmaeidae | *Brahmaea japonica* (Ishimori, 1924) | larval CNC with perinephric membrane |
|  |  |  |  |  |  | superfamily Geometroidea | | | | | *Phthonandria atrilineata* and *Abraxas sylvata* (Ishimori, 1924) | larval CNC with perinephric membrane |
|  |  |  |  |  |  | superfamily Drepanoidea | | | | | *Auzata superba* and *Oreta turpis* (Ishimori, 1924) | larval CNC with perinephric membrane |
|  |  |  |  |  |  | superfamily Noctuoidea | | | | | *Trichoplusia ni* (cabbage looper) (O'Donnell & Ruiz-Sanchez, 2015), multiple species (Ishimori, 1924) | larval CNC with perinephric membrane |
|  |  |  |  |  |  | superfamily Cossoidea | | | | | *Synanthedon hector* (Ishimori, 1924) | larval CNC with perinephric membrane |
|  |  |  |  |  |  | superfamily Zygaenoidea | | | | | *Monema flavescens* (Ishimori, 1924) | larval CNC with perinephric membrane |
|  |  |  |  |  |  | superfamily Papilionoidea | | | | family Hesperiidae | *Calpodes ethlius* (Brazilian skipper) (Irvine, 1969), *Parnara* *guttata* (Ishimori, 1924) | larval CNC with perinephric membrane |
|  |  |  |  |  |  |  |  |  |  | family Pieridae | *Pieris brassicae* (large white) (Ramsay, 1976), *Pieris rapae*, *Colias hyale* (Ishimori, 1924) | larval CNC with perinephric membrane |
|  |  |  |  |  |  |  |  |  |  | family Nymphalidae | *Bicyclus anynana* (squinting bush brown) (Ben King, unpublished observation), *Aglais urticae* (small tortoiseshell) (Henson, 1937), *Lethe sicelis*, *Vanessa indica*, *Hestina japonica* (Ishimori, 1924) | larval CNC with perinephric membrane |
|  |  |  |  |  |  |  |  |  |  | family Lycaenidae | *Taraka hamada* and *Polyommatus baeticus* (Ishimori, 1924) | larval CNC with perinephric membrane |
|  |  |  |  |  |  |  |  |  |  | family Papilionidae | *Papilio xuthus*, *P. machaon*, *P. demetrius* (Ishimori, 1924) | larval CNC with perinephric membrane |
|  |  | suborder Zeugloptera | | | family Micropterigidae | | | | | |  | no CNC (Barbehenn & Kristensen, 2003) |
|  |  | suborder Aglossata | | | family Agathiphagidae (kauri moths) | | | | | |  | no CNC (Barbehenn & Kristensen, 2003) |
|  |  | infraorder Exoporia | | | family Hepialidae (swift moths) | | | | | |  | no CNC (Barbehenn & Kristensen, 2003; Ishimori, 1924) |
|  | Diptera | family Keroplatidae (fungus gnats) | | | | | | | | | *Arachnocampa luminosa* (New Zealand glow worm) (Green, 1980; Wheeler & Williams, 1915) and *Arachnocampa flava* (Rigby & Merritt, 2011) | laterally displaced larval CNC with perinephric membrane and possibly leptophragmata (observed in larvae) |
|  |  |  |  |  |  |  |  |  |  |  | *Keroplatus testaceus* (Wheeler & Williams, 1915; Stammer, 1932) | larval rectal complex without perinephric membrane |
|  | Hymenop-tera | suborder Symphyta | | | | | | | | | 132 species of sawfly within the superfamilies Tenthredinoidea and Pamphilioidea (Maxwell, 1955) | larvae of some species have CNC with perinephric membrane, some with distal MpTs bound to rectum, some with no association of MpTs with rectum |
|  |  | family Formicidae | | | | | | | | | *Solenopsis saevissima* (fire ant) (Arab & Caetano, 2002) | rectal complex lacking perinephric membrane observed in adult workers |
|  | Neuropte-ra | family Myrmeleontidae (antlions) | | | | | | | | | *Acanthaclisis baetica* (Poll, 1936), *Myrmeleon formicarius* (Loziński, 1911), *Furgella intermedia* and *Palpares annulatus* (van Zyl & van der Linde, 2000). See also (Aspöck & Aspöck, 2007) | terrestrial larvae which have CNC with perinephric membrane |
|  |  |  |  |  |  |  |  |  |  |  | *Myrmeleon obscurus*, *Neuroleon* sp. and *Palpares* sp. (Quartey & Kumar, 1973) | CNC not observed in adults |
|  |  | family Ascalaphidae | | | | | | | | | *Ascalaphus africanus* (Quartey & Kumar, 1973) | CNC not observed in adults |
|  |  | family Nevrorthidae | | | | | | | | |  | aquatic larvae with no CNC (Beutel *et al*., 2010; Aspöck & Aspöck, 2007) |
|  |  | family Sisyridae (spongeflies) | | | | | | | | |  | aquatic larvae with single MpT associated with hindgut (Beutel *et al.*, 2010; Aspöck *et al*., 2001) |
|  | Thysano-ptera | family Thripidae | | | | | | | | | *Frankliniella occidentalis* (western flower thrips) (Dallai *et al*., 1991; Ullman *et al.*, 1989) | 2 of 4 MpTs have distal ends attached to rectal pads. No perinephric membrane. Observed in adult |
|  | Hemiptera | family Cicadellidae | | | | | | | | | *Hishimonus lamellatus* (leafhopper) (Dai *et al.*, 2019), *Tettigoniella cosmopolita* (Goodchild, 1988) | CNC ensheathed in fine perinephric membrane, perhaps for conservation of salts. Observed in adult |
|  |  | family Cicadidae | | | | | | | | | *Platypleura capitata* (cicada) (references within Goodchild, 1966, 1988) | CNC ensheathed in perinephric membrane, perhaps for conservation of salts |
|  |  | superfamily Cercopoidea | | | | | | | | | *Ptyelus flavescens* (frog hopper) (Goodchild, 1966, 1988) | CNC ensheathed in fine perinephric membrane, perhaps for conservation of salts |
| Diplopoda | Polyxeni-da | family Polyxenidae | | | | | | | | | *Polyxenus lagurus* (bristly millipede) (Schlüter & Seifert, 1985) | central region of MpTs in CNC-like structure with perinephric membrane. Observed in adult and juvenile stages |

**REFERENCES**

Arab, A. & Caetano, F. H. (2002). Segmental specializations in the Malpighian tubules of the fire ant Solenopsis saevissima Forel 1904 (Myrmicinae): an electron microscopical study. *Arthropod Structure & Development* **30**(4), 281-92.

Areekul, S. (1957). The Comparative Internal Larval Anatomy of Several Genera of Scarabaeidae (Coleoptera). *Annals of the Entomological Society of America* **50**(6), 562-577.

Aspöck, U. & Aspöck, H. (2007). Verbliebene Vielfalt vergangener Blüte. Zur Evolution, Phylogenie und Biodiversität der Neuropterida (Insecta: Endopterygota). *Denisia* **20**, 451-516.

Aspöck, U., Plant, J. D. & Nemeschkal, H. L. (2001). Cladistic analysis of Neuroptera and their systematic position within Neuropterida (Insecta: Holometabola: Neuropterida: Neuroptera. *Systematic Entomology* **26**(1), 73-86.

Baker, W. V. (1968). The gross structure and histology of the adult and larval gut of Pentalobus barbatus (Coleoptera: Passalidae). *The Canadian Entomologist* **100**(10), 1080-1090.

Barbehenn, R. V. & Kristensen, N. P. (2003). 6. Digestive and excretory systems. In *Teilband/Part 36 Vol 2: Morphology*, *Physiology*, *and Development*. (ed W. Kükenthal), pp. 165-188. De Gruyter, Berlin.

Bayon, C. (1981). Ultrastructure de l'epithelium intestinal et flore parietale chez la larve xylophage d'Oryctes nasicornis L. (Coleoptera : scrabaeidae). *International Journal of Insect Morphology and Embryology* **10**(5), 359-371.

Beaven, R., Denholm, B., Fremlin, M. & Scaccini, D. (2024*a*). Evidence for the independent evolution of a rectal complex within the beetle superfamily Scarabaeoidea. *bioRxiv (preprint)*.

Berberet, R. C. & Helms, T. J. (1972). Comparative Anatomy and Histology of Selected Systems in Larval and Adult Phyllophaga anxia (Coleoptera: Scarabaeidae). *Annals of the Entomological Society of America* **65**(5), 1026-1053.

Beutel, R. G., Friedrich, F. & Aspöck, U. (2010). The larval head of Nevrorthidae and the phylogeny of Neuroptera (Insecta). *Zoological Journal of the Linnean Society* **158**(3), 533-562.

Conet, M. A. (1934). Contribution à l 'histologie des tubes de Malpighi des Coléoptères Cryptonéphridiés. *Annales de la Société scientifique de Bruxelles* **54**, 189-200.

Dai, L., Yang, B., Wang, J., Zhang, Z., Yang, R., Zhang, T., Ren, Z. & Lin, C. (2019). The Anatomy and Ultrastructure of the Digestive Tract and Salivary Glands of Hishimonus lamellatus (Hemiptera: Cicadellidae). *Journal of Insect Science* **19**(4).

Dallai, R., Del Bene, G. & Marchini, D. (1991). The ultrastructure of Malpighian tubules and hindgut of Frankliniella occidentalis (Pergande) (Thysanoptera : Thripidae). *International Journal of Insect Morphology and Embryology* **20**(4), 223-233.

Dufour, L. (1834). Recherches anatomiques et considérations entomologiques sur quelques insectes coléoptères, compris dans les familles des Dermestins, des Byrrhiens, des Acanthopodes, et des Leptodactyles. *Annales des sciences naturelles Zoologie* **1**, 56-84.

Dufour, M. L. (1824). Recherches anatomiques sur les Carabiques et sur plusieurs autres insectes Coléoptères. *Annales des Sciences Naturelles* **Atlas des Tomes 1,2,3**, 27-42.

Dufour, M. L. (1840). Histoire des Métamorphoses et de l'Anatomie des Mordelles. *Annales des sciences naturelles* **14**, 225-240.

Dufour, M. L. (1842). Histoire comparative des métamorphoses et de l'anatomie des Cetonia aurata et Dorcus parallelipipedus. *Annales Des Sciences Naturelles* **18**, 162-181.

Edwards, E. E. (1930). On the Morphology of the Larva of *Dorcus parallelopipedus*, L. (Coleoptera). *Zoological journal of the Linnean Society* **37**(251), 93-108.

Fletcher, F. W. (1930). The Alimentary Canal of Phyllophaga Gracilis Burm. *The Ohio Journal of Science* **30**(2), 109-119.

Goodchild, A. (1988). Contributions to knowledge of water relations in hemiptera. ProQuest Dissertations Publishing.

Goodchild, A. J. P. (1966). Evolution of the alimentary canal in the Hemiptera. *Biological Reviews* **41**(1), 97-139.

Gorka, A. (1914). Experimentelle und morphologische Beitrfige zur Physiologic der Malpighi'schen Gefasse der Kafer. *Zoologische Jahrbücher. Abteilung für allgemeine Zoologie und Physiologie der Tiere* **34**, 233-338.

Green, L. F. B. (1980). Cryptonephric malpighian tubule system in a dipteran larva, the New Zealand glow- worm, Arachnocampa luminosa ( Diptera: Mycetophilidae): A structural study. *Tissue and Cell* **12**(1), 141-151.

Grimstone, A. V., Mullinger, A. M. & Ramsay, J. A. (1968). Further studies on the rectal complex of mealworm *tenebrio molitor*, L. (Coleoptera, Tenebrioidae). *Philosophical Transactions of the Royal Society of London. Series B*, *Biological Sciences* **253**(788), 343-382.

Gérard, P. (1942). Les tubes de Malpighi de la larve d'Oryctes Nasicornis L. *Annales de la Société royale zoologique de Belgique* **73**, 122-133.

Henson, H. (1937). The Structure and Post-Embryonic Development of Vanessa urticae (Lepidoptera).-11. The Larval Malpighian Tubules. *Proceedings of the Zoological Society* **B107**(1), 161-174.

Hinton, H. E. (1939). An inquiry into the natural classification of the Dryopoidea, based partly on a study of their internal anatomy. *Transactions of the Royal Entomological Society of London* **89**, 133-184.

Hinton, H. E. (1941). Notes on the internal anatomy and immature stages of M*ycetophagus quadripustulatus* (Linnaeus) (Coleoptera, Mycetophagidae). *Proceedings of the Royal Entomological Society of London. Series A*, *General Entomology* **16**, 39-48.

Irvine, H. B. (1969). Sodium and potassium secretion by isolated insect Malpighian tubules. *The American journal of physiology* **217**(5), 1520-1527.

Ishimori, N. (1924). Distribution of the Malpighian vessels in the wall of the rectum of lepidopterous larvae. *Annals of the Entomological Society of America* **17**, 75-86.

Ito, H. (1921). On the metamorphosis of the malpighian tubes of Bombyx Mori L. *Journal of Morphology* **35**, 195-211.

Ivie, M. A. (1985). Phylogenetic studies in the Bostrichiformia (coleoptera). The Ohio State University / OhioLINK.

Jones, C. R. (1940). The alimentary canal of Diplotaxis liberta Germ. (Scarabaeidae: Coleoptera). *The Ohio Journal of Science* **40**(2), 94-103.

Judy, K. (1968). *Studies on the metamorphosis of the alimentary canal of Hyalophora cecropia (L.)*, ProQuest Dissertations Publishing.

Khatib, S. M. H. (1946). Studies in GalerucinÆ. *Proceedings of the Indian Academy of Sciences - Section B* **23**(1), 1-38.

King, B. & Denholm, B. (2014). Malpighian tubule development in the red flour beetle (Tribolium castaneum). *Arthropod Structure & Development* **43**(6), 605-13.

Lison, L. (1938). Contribution à l'étude morphologique et histophysiologique du système malpighien de Melolontha melolontha Linn. (Coleoptera). *Annales de la Société royal zoologique de Belgique* **69**, 195-233.

Loziński, P. (1911). Über die Malpighischen Gefäße der Myrmeleoniden als Spinndrüsen. *Zoologischer Anzeiger* **38**, 401-417.

Machin, J. & O'Donnell, M. J. (1991). Rectal complex ion activities and electrochemical gradients in larvae of the desert beetle, Onymacris: Comparisons with Tenebrio. *Journal of Insect Physiology* **37**(11), 829-838.

Maxwell, D. E. (1955). The Comparative Internal Larval Anatomy of Sawflies (Hymenoptera: Symphyta). *Memoirs of the Entomological Society of Canada* **87**(S1), 5-132.

McKenna, D. D., Shin, S., Ahrens, D., Balke, M., Beza-Beza, C., Clarke, D. J., Donath, A., Escalona, H. E., Friedrich, F., Letsch, H., Liu, S., Maddison, D., Mayer, C., Misof, B., Murin, P. J., Niehuis, O., Peters, R. S., Podsiadlowski, L., Pohl, H., Scully, E. D., Yan, E. V., Zhou, X., Ślipiński, A. & Beutel, R. G. (2019). The evolution and genomic basis of beetle diversity. *Proceedings of the National Academy of Sciences of the United States of America* **116**(49), 24729-24737.

Menees, J. H. (1958). The Anatomy and Histology of the Larval Alimentary Canal of the European Chafer, Amphimallon Majalis Razoumowsky (Scarabaeidae). *Journal of the New York Entomological Society* **66**(1/2), 75-86.

Metalnikov, S. (1908). Recherches expérimentales sur les Chenilles de Galleria mellonella  *Archives de zoologie expérimentale et générale* **4**, 489-588.

Mobüsz, A. (1897). Ueber den Darmkanal der Anthrenus-larve nebst Bemerkungen zur Epithel regeneration. *Archiv für Naturgeschichte* **63**, 89-128.

Nagae, T., Miyake, S., Kosaki, S. & Azuma, M. (2013). Identification and characterisation of a functional aquaporin water channel (*Anomala cuprea* DRIP) in a coleopteran insect. *The Journal of Experimental Biology* **216**(14), 2564-2572.

O'Donnell, M. J. & Ruiz-Sanchez, E. (2015). The rectal complex and Malpighian tubules of the cabbage looper (Trichoplusia ni): regional variations in Na+ and K+ transport and cation reabsorption by secondary cells. *The Journal of Experimental Biology* **218**(20), 3206.

Poll, M. (1932). Note sur la fonction des tubes de Malpighi des coléoptères. *Bulletin & annales de la Société entomologique de Belgique* **72**, 103-109.

Poll, M. (1936). Contribution à l’étude du l’histophysiologie de l’appareil urinaire des larves de Myrmeleontides. *Mémoires du Musée royal d'histoire naturelle de Belgique* **3**, 636-666.

Poyarkoff, E. (1910). Recherches histologiques sur la métamorphose d’un coléoptère (la galéruque de l’orme). *Archives d'anatomie microscopique et de morphologie experimentale* **12**, 333-474.

Pradhan, S. (1942). Re-association of Malpighian tubules in coccinellid beetles. *The Indian Journal of Entomology* **4**, 11-21.

Quartey, S. Q. & Kumar, R. (1973). Structure of the Alimentary and Reproductive Organs of Some Adult Neuroptera. *Entomalogica Scandinavica* **4**, 91-99.

Ramsay, J. A. (1964). The rectal complex of the mealworm *Tenebrio molitor*, L. (Coleoptera, Tenebrionidae). *Philosophical Transactions of the Royal Society of London. Series B*, *Biological Sciences* **248**(748), 279-314.

Ramsay, J. A. (1976). The rectal complex in the larvae of Lepidoptera. *Philosophical Transactions of the Royal Society of London. B*, *Biological Sciences* **274**(932), 203-226.

Rigby, L. M. & Merritt, D. J. (2011). Roles of biogenic amines in regulating bioluminescence in the Australian glowworm *Arachnocampa flava*. *The Journal of Experimental Biology* **214**(19), 3286-3293.

Saini, R. S. (1964). Histology and physiology of the cryptonephridial system of insects. *Transactions of the Royal Entomological Society of London* **116**(14), 347-392.

Schlüter, U. & Seifert, G. (1985). Functional morphology of the hindgut-malpighian tubulecomplex in Polyxenus lagurus (Diplopoda; Penicillata). *Bijdragen tot de Dierkunde* **55**(1), 209-218.

Schäfer, R. (1954). Zur Kenntnis der Anatomie, Physiologie und Ökologie des Brachkäfers, Rhizotrogus aestivus Oliv. (Col. Lam.). *Zeitschrift für Angewandte Entomologie* **35**(4), 381-424.

Sheehan, C. M., Crawford, A. M. & Wigley, P. J. (1982). Anatomy and histology of the alimentary canal of the black beetle, Heteronychus arator. *New Zealand Journal of Zoology* **9**(3), 381-385.

Sirodot, M. S. (1858). Recherches sur les sécrétions chez les insects. *Annales des sciences naturelles* **10**, 141-189.

Stammer, H.-J. (1932). Zur biologie und anatomie der leuchtenden pilzmückenlarve von ceroplatus testaceus dalm. (Diptera, Fungivoridae). *Zeitschrift für Morphologie und Ökologie der Tiere* **26**(1), 135-146.

Stammer, H.-J. (1934). Bau und bedeutung der malpighischen gefässe der coleopteren. *Zeitschrift für Morphologie und Ökologie der Tiere* **29**(1), 196-217.

Swingle, M. (1930). Anatomy and Physiology of the Digestive Tract of the Japanese Beetle: Illus. *Journal of Agricultural Research* **41**, 181.

Ullman, D. E., Westcot, D. M., Hunter, W. B. & Mau, R. F. L. (1989). Internal anatomy and morphology of Frankliniella occidentalis (Pergande) (Thysanoptera: Thripidae) with special reference to interactions between thrips and tomato spotted wilt virus. *International Journal of Insect Morphology and Embryology* **18**(5), 289-310.

van Zyl, A. & van der Linde, T. C. K. (2000). Anatomy and histology of the alimentary canals of the antlion larvae F*urgella intermedia* Markl and P*alpares annulatus S*titz (Neuroptera: Myrmeleontidae), with reference to their feeding physiology. *African Entomology* **8**, 179-188.

Verma, P. S. (1969). The alimentary canal and associated organs of two saprophagous beetle, Onthophagus catta Fabr. and Aphodius moestus Fabr. (Coleoptera: Scarabaeidae). *Bulletin of entomology* **10**(1), 4-11.

Wang, M.-J., Sun, X.-Y. & Jiang, L. (2024). Ultrastructural comparison of the larval midguts between *Trypoxylus dichotomus* (Linnaeus, 1771) and *Anomala corpulenta* (Motschulsky, 1854) (Coleoptera: Scarabaeidae). *Tissue and Cell* **90**.

Werner, E. (1926). Die ernährung der larve von potosia cuprea fbr. (Cetonia floricola hbst.). *Zeitschrift für Morphologie und Ökologie der Tiere* **6**(1), 150-206.

Wheeler, W. M. & Williams, F. X. (1915). The Luminous Organ of the New Zealand Glow-worm. *Psyche: A Journal of Entomology* **22**(2), 36-43.

Wigglesworth, V. B. (1974*b*). *The principles of insect physiology*, Seventh edition.. edition. London : Chapman and Hall, London.

Woods, W. C. (1916). The Malpighian Vessels of Haltica Bimarginata Say (Coleoptera). *Annals of the Entomological Society of America* **9**(4), 391-406.
